# *ATP1A3* mosaicism in families with alternating hemiplegia of childhood

**DOI:** 10.1111/cge.13539

**Published:** 2019-04-03

**Authors:** Xiaoling Yang, Xiaoxu Yang, Jiaoyang Chen, Shupin Li, Qi Zeng, August Y. Huang, Adam Y. Ye, Zhe Yu, Sheng Wang, Yuwu Jiang, Xiru Wu, Qixi Wu, Liping Wei, Yuehua Zhang

**Affiliations:** ^1^ Department of Pediatrics Peking University First Hospital Beijing China; ^2^ Center for Bioinformatics, State Key Laboratory of Protein and Plant Gene Research, School of Life Sciences Peking University Beijing China; ^3^ Academy for Advanced Interdisciplinary Studies Peking University Beijing China; ^4^ Peking‐Tsinghua Center for Life Sciences Peking University, Beijing China; ^5^ Human Genetic Resources Core Facility, School of Life Sciences Peking University Beijing China; ^6^ Dr Liping Wei's lab, National Institute of Biological Sciences Beijing China; ^7^ College of Biological Sciences China Agricultural University Beijing China

**Keywords:** alternating hemiplegia of childhood, *ATP1A3*, *de novo*, micro‐droplet digital PCR, mosaicism

## Abstract

Alternating hemiplegia of childhood (AHC) is a rare and severe neurodevelopmental disorder characterized by recurrent hemiplegic episodes. Most AHC cases are sporadic and caused by *de novo ATP1A3* pathogenic variants. In this study, the aim was to identify the origin of *ATP1A3* pathogenic variants in a Chinese cohort. In 105 probands including 101 sporadic and 4 familial cases, 98 patients with *ATP1A3* pathogenic variants were identified, and 96.8% were confirmed as *de novo*. Micro‐droplet digital polymerase chain reaction was applied for detecting *ATP1A3* mosaicism in 80 available families. In blood samples, four asymptomatic parents, including two paternal and two maternal, and one proband with a milder phenotype were identified as mosaicism. Six (7.5%) parental mosaicisms were identified in multiple tissues, including four previously identified in blood and two additional cases identified from paternal sperms. Mosaicism was identified in multiple tissues with varied mutant allele fractions (MAFs, 0.03%‐33.03%). The results suggested that MAF of mosaicism may be related to phenotype severity. This is the first systematic report of *ATP1A3* mosaicism in AHC and showed mosaicism as an unrecognized source of previously considered “*de novo*” AHC. Identifying *ATP1A3* mosaicism provides more evidence for estimating recurrence risk and has implications in genetic counseling of AHC.

## Graphical abstract

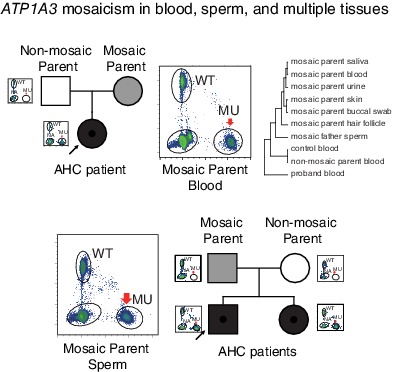

## 1 INTRODUCTION

Alternating hemiplegia of childhood (AHC, MIM: 614820) is a rare and predominantly sporadic neurological disorder characterized by attacks of hemiplegia and other paroxysmal manifestations such as abnormal eye movement and dystonia.^1^ Additional symptoms may appear later in the disease process.^2^ Several study groups have identified *de novo* pathogenic variants in the *ATP1A3* gene as the cause of AHC in 93.3% to 100% of patients.^3^, ^4^, ^5^

*De novo* variants have been discovered to be a more prominent disease‐causing mutation type than inherited variants in numerous neurological genetic disorders, such as Dravet syndrome and autism spectrum disorders.^6^, ^7^ *De novo* variant was an alteration in a gene that is present for the first time in one family member as a result of a mutation in a germ cell (egg or sperm) of one of the parents or in the fertilized egg itself. *De novo* variants are detectable in the disease‐affected probands but undetectable in either of their parents in genomic DNA from peripheral blood sample. The consistency of gene‐associated phenotypes and patients' manifestations were confirmed clinically.^8^ Many variants were considered *de novo* by either direct Sanger sequencing or next‐generation sequencing (NGS) results validated by Sanger sequencing.^3^, ^4^, ^5^ *De novo* variants are considered to be either mainly prezygotic variants in the germ cells of parents, or postzygotic variants in the offspring.^9^, ^10^ They have been reported to have strong paternal origin bias in large‐scale family data.^11^, ^12^, ^13^ Some postzygotic mosaicisms in the parents may occur at the early stage and affect both the germ cells and somatic cells, known as gonosomal mosaicism.^14^ Recent publications have further reported that some “*de novo*” pathogenic variants are actually inherited from underestimated parental mosaicism.^15^, ^16^

In AHC, *de novo* single nucleotide pathogenic variants are more common than inherited variants.^3^, ^4^, ^5^ However, few families with autosomal dominant inheritance have been reported. A family with two AHC half‐sisters born to an asymptomatic mother and different fathers, suggesting maternal mosaicism.^17^ Hully *et al* reported two unrelated families with two full siblings with *ATP1A3* variants presenting epilepsy and ataxia, and the authors suggested the potential occurrence of parental *ATP1A3* mosaicism.^18^

More sensitive genetic testing approaches are required for the detection and validation of mosaicism in AHC‐affected families with *ATP1A3* variants, especially in parents with multiple AHC‐affected children. NGS‐based deep sequencing methods, such as personal genome machine amplicon sequencing for mosaicism (PASM) and molecular inversion probes, can be used to detect mutant allele fraction (MAF) as low as 1%, and duplex sequencing can detect even lower percentages.^19^ However, deep sequencing methods targeting the whole exome or genome require significantly increased sequencing depth to achieve higher detection limits. Next‐generation micro‐droplet digital polymerase chain reaction (mDDPCR) can be used to detect MAF as low as 0.01%, as is suitable for hotspot variants.^20^

Here, we first report parental *ATP1A3* mosaicism in AHC families, regarded as “*de novo*” via Sanger sequencing, by mDDPCR and PASM using blood, sperm, and other tissues. We also identified *ATP1A3* mosaicism in one proband with milder AHC.

## 2 MATERIALS AND METHODS

### 2.1 Patient recruiting and inclusion criteria

AHC patients were recruited from Peking University First Hospital from August 2005 to May 2016. This study and the methodologies involved were in accordance with the relevant guidelines and regulations of the Institutional Review Board at Peking University (IRBPU) under the approval IRB00001052‐11087. Written informed consent was provided by the patients or their statutory guardians. We labeled each family with Axxx according the enrolling time. In each family, each person has separate IDs. For example, A052 was the family ID. In this family, each family member was assigned a separate ID as: A05203: proband; A05201: father and A05202: mother.

**Figure 1:**
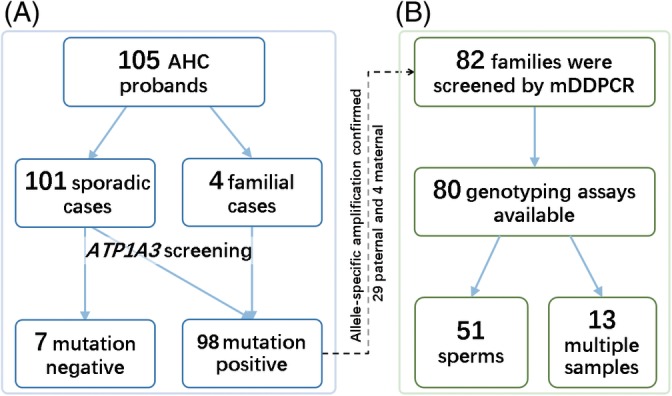
Schematic flowchart of patient recruiting and screening procedures. A, Schematic flowchart for patient recruiting and variant screening procedures. B, TaqMan MGB genotyping assays could be designed to perform variant allele discrimination in available families [Colour figure can be viewed at http://wileyonlinelibrary.com]

All typical patients fulfilled the published clinical diagnostic criteria for AHC^1^, ^2^: (a) onset of first symptoms before 18 months of age; (b) repeated episodes of hemiplegia involving alternating body sides; (c) episodes of bilateral hemiplegia or quadriplegia, starting bilaterally as a separate attack or as a generalization of a hemiplegic episode; (d) disappearance of all symptoms upon sleeping, and symptoms possibly resuming later after waking; (e) other paroxysmal disturbances, including dystonic spells, abnormal oculomotor symptoms, or autonomic phenomena, occurring during hemiplegic spells or independently; and (f) evidence of developmental delay or neurologic abnormalities, including choreoathetosis, dystonia, or ataxia. Atypical cases with onset age later than 18 months but who fulfilled all other diagnostic criteria for AHC were also included.^21^, ^22^ A Chinese AHC cohort consisting of 105 probands, 101 sporadic and 4 familial, were recruited (Figure 1A). Fifty‐one patients had been described in our previous publication,^5^ and the remaining patients were not reported. The four multiplex families included one dominant family with two affected family members, two sets of monozygotic twins who were both affected with AHC, and one family with sibship of an affected brother and sister.

### 2.2 DNA isolation, *ATP1A3* variant screening, and functional prediction of variants

Genomic DNA from peripheral blood was extracted using a salting‐out procedure. Genomic DNA from buccal swab, saliva, hair follicles and urine was extracted using a QIAamp DNA Micro Kit (#56304, Qiagen, Germany). Skin biopsies were collected from a 5‐mm‐diameter skin punch, and DNA was extracted using an All Prep DNA/RNA Mini Kit (#80204; Qiagen). Sperm samples were purified with a PureSperm 40/80 assay (Nidacon, Sweden). Genomic DNA of purified sperm cells and unpurified semen were extracted using a phenol‐chloroform extraction method.^15^

*ATP1A3* variants were screened by Sanger sequencing or panel NGS. Before 2015, 74 patients were screened by Sanger sequencing. After 2015, the other 31 patients were sequenced by NGS panel sequencing. A total of 153 genes including *ATP1A3* were included in the panel and listed in Supporting Information, Table S1. All 23 coding exons and their flanking regions were sequenced using primers as previously described.^4^ Variants described in this article were based on reference cDNA NM_152296.4, reference amino acid sequence NP_689509.1, and reference genome assembly hg19. Deleterious variants detected in the probands were then sequenced in the blood samples of their parents or other available family members. Hundred unrelated healthy individuals were sequenced as controls. Functional predictions of the variants were carried out using conventional prediction software as well as the classifiers we developed for AHC or *ATP1A3*.^5^, ^23^ The variant and phenotype information have been deposited in https://databases.lovd.nl/shared/genes/*ATP1A3*.

### 2.3 Detection of mutant allele origin and paternity

Allele‐specific PCR (ASPCR) was carried out to determine the origins of the mutant alleles in the probands. Primers for amplifying the SNP fragments are listed in Table S2. For ASPCR, one or two pairs of allele‐specific primers were designed based on the informative SNPs. The patients' two alleles were analyzed separately using paternal or maternal‐specific primers containing different bases of the target variants. The primers used for ASPCR are listed in Table S3.

Short tandem repeat (STR) analysis was carried out for all informative families to confirm paternity and gender using 11 highly polymorphic microsatellite markers. The forward primer of each pair was labeled with FAM fluorophores. Primers for STR analysis are provided in Table S4. Labeled amplicons were detected on an ABI 3730 automated sequencer (Applied Biosystems by Thermo Fisher) and analyzed using genotype software (Gene Marker 1.5; SoftGenetics).

### 2.4 mDDPCR screening and PASM for mosaicism

Raindrop mDDPCR was used for the detection of low‐fraction mosaicism in 80 available families (Figure 1B). Genotyping assays were first tested using an endpoint‐genotyping experiment. Ultraviolet treatments were carried out after each reaction to reduce contamination. After droplet emulsion generation and PCR reactions, post‐PCR emulsions were subjected to a droplet detector, and data were analyzed using RainDrop Analyst V3 software (RainDance Technologies, Billerica, Massachusetts). A binominal distribution was fitted, and the 95% confidence intervals (CIs) of MAFs were calculated. If the lower bound of the 95% CI of the MAF detected by mDDPCR was >0.01% and the upper bound was <50.00%, it was considered to be a positive detection according to our previous benchmark test.^15^ MAFs in detectable *ATP1A3* mosaic families were further quantified using PASM. The primers of PASM detection were provided in Table S5.

### 2.5 Prenatal diagnosis

Prenatal diagnosis was performed upon further pregnancy of one proband's mother at 22 gestational weeks. A 20‐mL amniotic fluid sample was collected under ultrasound guidance and cultured before DNA extraction. Fetal DNA was extracted using a Wizard Genomic DNA Purification Kit (Promega) according to the manufacturer's instructions. Before DNA sequencing, maternal cell contamination was excluded by STR analysis, as described above. The sex of the fetus was determined by karyotype analysis and PCR amplification of the sex‐determining region Y (NM_003140).

## 3 RESULTS

### 3.1 Sanger screening of *ATP1A3* pathogenic variants in probands and parents

**Figure 2:**
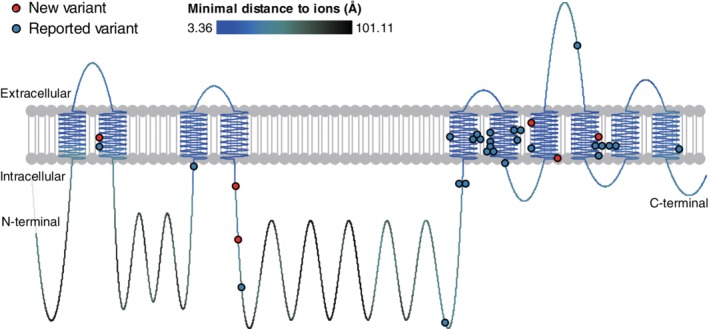
Locations of *ATP1A3* variants shown on protein domains. Thirty‐three variants were identified. Six newly identified *ATP1A3* variants (red dots) and variants reported previously (blue dots) are shown [Colour figure can be viewed at http://wileyonlinelibrary.com]

Thirty‐three deleterious variants were identified in the blood samples from 98 (93.3%, 98/105) probands (Table S6 and Figure 2). Of the 33 variants, 6 were novel. None of the 33 variants were common SNPs in dbSNP 137, and they were all absent in 1000 Genomes, the Personal Genome Project, ESP6500, gnomAD or ExAC. Their functional effects were predicted by Polyphen2, iFish and our logistic classifier (Table S6). The predicted functional effects for variants from this study and reported causal variants from the literature are similar, and these were different from the benign missense variant in the *ATP1A3* coding region in ExAC (Figures S1 and S2). According to the ACMG standard and guidelines,^24^ all variants were classified as likely pathogenic.

**Figure 3:**
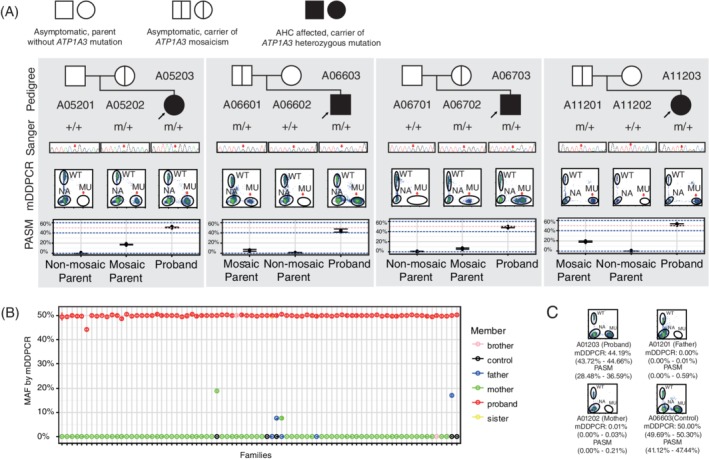
mDDPCR and PASM detected *ATP1A3* variants from blood samples of 80 AHC families. A, Four parental mosaic variants were detected by mDDPCR, and their pedigree charts are shown. Mosaic variants are clearly showed on the flow cytometry scatter plots of mDDPCR under the cluster name of “MU” at the bottom right corner. Peripheral blood from the proband was used as a positive control; the performance of the negative controls is not shown. B, mDDPCR considered AHC‐affected families with “*de novo*” *ATP1A3* variants. Probands were all detected with a mutant allelic fraction (MAF) of 50%. The lower bounds of the 95% binominal CIs of the measured MAFs were under 0.01% in the non‐mosaic parents and negative controls. C, The MAF identified by mDDPCR and PASM confirmed that A01203 carried a pathogenic variant on c.2839G>C/p.(Gly947Arg) with the MAF and the 95% confidence intervals that met the detection criteria for a mosaic variant. The proband A06603, who was detected with a heterozygous variant at the same base c.2839G>C/p.(Gly947Arg), was chosen as a positive control [Colour figure can be viewed at http://wileyonlinelibrary.com]

For 93 out of 98 probands with *ATP1A3* variants, Sanger sequencing was performed in the blood DNA of their parents. We identified 96.8% (90/93) as *de novo* pathogenic variants and 3.2% (3/93) as inherited. For the three patients with inherited variants, one patient inherited the heterozygous variant from her AHC‐affected mother,^5^ and the other two patients inherited the variant from the asymptomatic mother (A05202) or father (A11201), suggesting the mosaicism due to the lower signal (Figure 3A). ASPCR amplification were performed in 33 families with available SNPs. Twenty‐nine (87.9%) were paternal and four (12.1%) were maternal, indicating significant parent‐of‐origin sex bias. One index case of paternal origin is shown in Figure S3, and the variants and parental origins are listed in Table S7.

In the family with two affected siblings (Family A065, Figure S4), the brother and sister carried the same pathogenic variant c.2401G>A/p. (Asp801Asn), and no variant signals were detected from the parents' blood DNA by Sanger sequencing. ASPCR amplification confirmed that both the proband and his sister inherited the variant from their father (Figure S5).

### 3.2 Mosaic variants were detected by mDDPCR in the blood of four parents and one atypical proband

Among AHC families that were considered to carry “*de novo*” or “parental mosaic” *ATP1A3* pathogenic variants by Sanger sequencing, 82 families agreed to participate to further genetic tests of mosaicism screening, including Family A052, A112 and A065. Twenty‐four genotyping assays were available for 80 families according to an end‐point genotyping qPCR analysis (Figure S6 and S7).

Four of 80 (5%) parental mosaic families were detected from the peripheral blood samples by mDDPCR. The MAFs were quantified and validated as 18.82% in the mother of Family A052, 17.05% in the father of Family A112, 7.65% in the mother of Family A067 and 7.53% in the father of Family A066. The MAFs of PASM were similar to those obtained by mDDPCR (Figure 3A and Table 1).

**Table 1:** Positive mosaic families with blood samples of different origins (MAF and 95% CI in %)

Variant info								Father blood		Mother blood		Control blood		Proband blood	
Family	Origin	Chr	Position^a^	Ref	Alt	Var (cDNA)^b^	Var(protein)^c^	mDDPCR	PASM	mDDPCR	PASM	mDDPCR	PASM	mDDPCR	PASM
A012	Proband	19	42471896	C	G	c.2839G>C	p.(Gly947Arg)	0.00 (0.00‐0.01)	0.0 (0.0‐0.6)	0.01 (0.00‐0.03)	0.0 (0.0‐0.2)	0.00 (0.00‐0.02)	0.0 (0.0‐0.2)	44.19 (43.72‐44.66)	32.5 (28.5‐36.6)
A052	Maternal	19	42473598	C	T	c.2677G>A	p.(Gly893Arg)	0.00 (0.00‐0.01)	0.0 (0.0‐0.0)	18.82 (18.51‐19.14)	17.1 (16.7‐17.6)	0.00 (0.00‐0.02)	0.0 (0.0‐0.0)	49.98 (49.60‐50.35)	50.9 (49.9‐51.9)
A066	Paternal	19	42471896	C	G	c.2839G>C	p.(Gly947Arg)	7.53 (7.33‐7.74)	4.5 (2.8‐6.7)	0.01 (0.00‐0.02)	0.0 (0.0‐0.4)	0.00 (0.00‐0.02)	0.0 (0.0‐0.2)	50.00 (49.69‐50.30)	44.3 (41.1‐47.4)
A067	Maternal	19	42471896	C	T	c.2839G>A	p.(Gly947Arg)	0.00 (0.00‐0.01)	0.2 (0.0‐1.3)	7.65 (7.40‐7.91)	6.4 (5.7‐7.2)	0.00 (0.00‐0.01)	0.0 (0.0‐0.2)	50.41 (49.88‐50.95)	49.2 (48.0‐50.5)
A112	Paternal	19	42486187_42486189	GAA		c.1063_1065del	p.(Glu355del)	17.05 (16.82‐17.28)	18.8 (17.7‐19.9)	0.00 (0.00‐0.01)	0.0 (0.0‐0.1)	0.0 (0.00‐0.01)	NA	49.93 (49.64‐50.22)	52.6 (50.7‐54.6)

Abbreviations; CI, confidence interval; MAF, mutant allele fraction; mDDPCR, micro‐droplet digital polymerase chain reaction; NA, not available.

a Genomic position based on hg19.

b All variants based on cDNA NM_152296.4.

c Reference amino acid sequence NP_689509.1.

Mosaicism was also confirmed by mDDPCR and PASM in an atypical AHC proband (A01203) carrying the hotspot variant c.2839G>C/p.(Gly947Arg). MAF measured using the blood sample of proband A01203 was 44.19% by mDDPCR and 32.5% by PASM, which was significantly lower than all the other probands (Figure 3B,C). The onset age and the age of first hemiplegia of A01203 were distinctly later. The clinical phenotype of the A01203 was milder, compared with the four probands carrying the same heterozygous variant (Table 2).

**Table 2:** Phenotype of probands carrying the p.(Gly947Arg) pathogenic variant

	Patients ID				
	A01203	A02903	A05503	A06603	A08203
Sex	M	F	M	M	M
Father	−	−	−	−	−
Mother	−	+	−	−	−
Age of onset (months)	30	5	7	5	3
Abnormal eye movement	−	−	−	+	−
Dystonia	−	+	+	−	+
Hemiplegia	+	−	+	−	−
Quadriplegia	−	−	−	−	−
Seizure	−	−	−	−	−
Hemiplegia start (months)	30	11	7	8	3
Hemiplegia duration (days)	0.01~4	2~7	0.01~3	1~2	2~3
Hemiplegia frequency (per month)	3~15	1~2	2~3	2	2~3
Quadriplegia	Uncertain	+	+	−	−
Abnormal eye movement	+	+	−	+	−
Dystonia	+	+	+	+	+
Epilepsy	−	−	−	−	−
Automatic dysfunction	−	−	−	−	−
Dysphagia	+	−	−	−	−
Dysarthria	+	−	−	−	−
Respiratory disturbance	−	−	−	−	−
Ataxia	−	Uncertain	+	+	−
Developmental delay	+	+	+	+	Uncertain

Abbreviations: F, female; M, male

### 3.3 Two germline mosaic variants were only detected in paternal sperm samples

**Figure 4:**
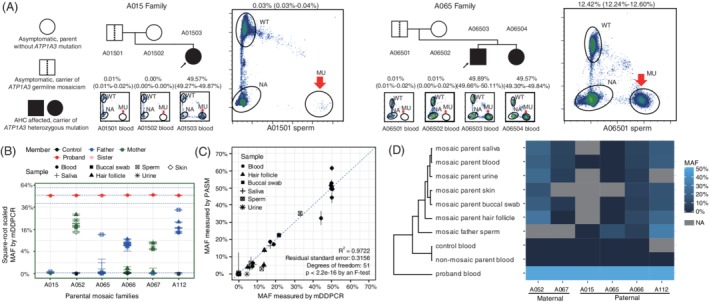
Screen of multiple parental peripheral samples and paternal sperm samples identified two additional mosaic cases. A, Mutant alleles were identified from the sperm samples of the A015 and A065 fathers (A01501 and A06501, sperm samples are enlarged); no signal that could pass the threshold was identified from their blood samples. MAFs and 95% binomial confidence intervals (CIs) are labeled for each sample. B, Mosaic variants are detectable from multiple parental peripheral samples. Error bars show the 95% CIs from binomial estimations for MAFs. Shapes are used to distinguish tissues, and colors distinguish family members. MAFs of A01501 sperm were difficult to distinguish from 0. C, The multiple parental samples were also examined by PASM, and the correlation of the PASM and mDDPCR‐estimated MAFs is shown. D, Hierarchical clustering for square‐root‐transformed MAFs measured by mDDPCR from multiple parental peripheral samples is shown using Euclidean distance measurement. Mosaic parental urine samples clustered closer to the branch of non‐mosaic control samples than other mosaic parental samples. Blood and saliva MAFs clustered closer than other parental samples. Skin punch and hair follicle samples showed similar MAFs. Details for the mDDPCR analysis for parental tissues are shown in Figures S8 and S9 [Colour figure can be viewed at http://wileyonlinelibrary.com]

Given the high frequency of the paternal origin of the mutant alleles, we speculated that a collection of paternal sperm and multiple parental peripheral tissues might provide further evidence for parental mosaicism and important information for genetic counseling. Sperm samples from 51 fathers were collected (Table S8), including parental mosaic cases, and healthy sperm was purified and subjected to mDDPCR. Apart from the mosaicisms that were detectable in paternal blood, mosaicisms were detected in sperms of two additional cases, A01501 (MAF of 0.03%) and A06501 (MAF of 12.42%; Figure 4A,B). Taken together, six (7.5%) parental mosaic cases were identified from the *ATP1A3* mutated AHC affected families.

### 3.4 Mosaic variants were detected in multiple peripheral samples

Multiple samples from parents, including buccal swab, saliva, hair follicles, skin punch and urine, were collected from 13 individuals from 11 AHC families. Among the 13 individuals, 6 parents were from five mosaic families (A052, A065, A066, A067, and A112) and the other 7 parents were from six non‐mosaic families (A012, A023, A050, A061, A082 and A111). Mosaic variants were also detected in multiple samples from blood‐positive mosaic parents (Figure 4B and Table 3), but with different MAF values. Heterogeneity of the MAFs was observed among the different tissues, and the shared mosaicism suggested that the variants might have occurred at the early stages of embryonic development. High correlation was observed between MAFs measured by PASM and mDDPCR (*R* ^2^ = 0.9722; Figure 4C), showing that the differences of MAF we observed are reliable.

**Table 3:** Positive mosaic families with multiple tissue samples of different origins (MAF and 95% CI in %)

Variant info								Father sperm	Mosaic parents' multiple peripheral samples					
Family	Origin	Chr	Position^a^	Ref	Alt	Var (cDNA)^b^	Var(protein)^c^		Blood	Buccal swab	Saliva	Hair follicle	Urine	Skin punch
A015	Paternal	19	42474436	C	T	c.2443G>A	p.(Glu815Lys)	0.03 (0.03‐0.04)	0.01 (0.01‐0.02)	NA	NA	NA	NA	NA
A052	Maternal	19	42473598	C	T	c.2677G>A	p.(Gly893Arg)	NA	18.82 (18.51‐19.14)	17.60 (17.19‐18.02)	20.51 (20.27‐20.75)	28.25 (27.58‐28.93)	23.24 (22.48‐24.01)	14.13 (13.64‐14.62)
A065	Paternal	19	42474557	C	T	c.2401G>A	p.(Asp801Asn)	12.42 (12.24‐12.60)	0.01 (0.01‐0.02)	0.01 (0.00‐0.04)	0.01 (0.00‐0.01)	0.00 (0.00‐0.98)	0.00 (0.00‐1.73)	NA
A066	Paternal	19	42471896	C	G	c.2839G>C	p.(Gly947Arg)	6.41 (6.26‐6.56)	7.53 (7.33‐7.74)	6.48 (6.34‐6.62)	8.05 (7.82‐8.29)	9.64 (9.39‐9.90)	5.49 (4.17‐7.09)	7.70 (7.48‐7.93)
A067	Maternal	19	42471896	C	T	c.2839G>A	p.(Gly947Arg)	0.00 (0.00‐0.00)	7.65 (7.40‐7.91)	4.87 (4.52‐5.23)	8.08 (7.90‐8.27)	0.05 (0.00‐0.25)	5.90 (4.80‐7.16)	NA
A112	Paternal	19	42486187_42486189	GAA		c.1063_1065del	p.(Glu355del)	33.03 (32.78‐33.27)	17.05 (16.82‐17.28)	13.88 (13.30‐14.49)	16.92 (16.65‐17.18)	21.66 (21.37‐21.95)	0.00 (0.00‐0.00)	14.03 (13.59‐14.49)

Abbreviation: CI, confidence interval; MAF, mutant allele fraction; NA, not available.

a Genomic position based on hg19.

b All variants based on cDNA NM_152296.4.

c Reference amino acid sequence NP_689509.1.

To analyze the MAF differences among multiple samples, we performed hierarchical clustering for square‐root‐transformed MAFs detected by mDDPCR from the parental samples (Figure 4D). Euclidean distances were used for comparisons. From the clustering result, the probands showed the lowest similarity to the other samples. Samples from the blood of normal controls and non‐mosaic parents had higher similarities. Most samples from mosaic parents showed a similar pattern. MAFs in blood and saliva were the most similar because of their mesodermal origin. Buccal swabs containing ectoderm‐derived oral epithelium as well as mesoderm‐derived white blood cells showed a similar MAF to the skin punch. These findings showed that the cell type might be an important factor influencing MAF. Additional cell divisions from the common progenitor cells might also contribute to this phenomenon. The mDDPCR raw flow cytometry scatter plots are presented in Figure S8, and MAF differences are compared in Figure S9.

### 3.5 Prenatal diagnosis in families with parental *ATP1A3* mosaicism

Prenatal tests were then carried out for the mother from Family A067 c.2839G>A/p.(Gly947Arg) who was pregnant and expressed worries of AHC recurrence in the fetus. The mother was a mosaic carrier of the same *ATP1A3* pathogenic variants as her first child. The MAFs measured 7.65% from blood, 8.08% from saliva, 4.87% from buccal swab, 5.9% from urine and 0.05% from hair follicle (Table 3), suggesting a higher risk of recurrence. DNA of the fetus was extracted from the amniotic tissues, and Sanger sequencing was carried out to detect the same *ATP1A3* pathogenic variants of the proband. No detectable mutant signals were obtained in the fetus (Figure S10).

## 4 DISCUSSION

In our Chinese cohort of 101 sporadic cases and 4 familial cases, 33 *ATP1A3* pathogenic variants were identified in 93.3% of the AHC patients. Of these, 96.8% were identified as *de novo* variants. We identified six (7.5% of 80 families, 4/80 in peripheral blood and 2/51 in sperm) mosaicism in the asymptomatic parents and one mosaic variant in the proband with milder phenotype by mDDPCR and PASM.

Sanger sequencing could only detect mosaicism with an MAF higher than 10%. For A05202 and A11201, the variant signal highly suggested the mosaicism and MAF of 18.82% and 17.05% was validated with mDDPCR. However, the low‐level MAF could not be detected using conventional Sanger sequencing or low‐coverage NGS. For A06601 and A06702, the Sanger sequencing result failed to tell the mosaicism and MAF of 7.53% and 7.65% were detected by mDDPCR. More importantly, even for whole‐exome data with an average depth of 200×, it was relatively difficult to determine mosaicism with MAF ~1%. For the majority of mosaic variants with a lower MAF, only more sensitive detection techniques could identify and quantify the mosaicism. From our benchmark tests and multiple validation of ultra‐low fraction mosaicism, PASM (with an average sequencing depth of ~10 000×) could detect MAF as low as 0.5%,^15^, ^25^, ^26^ and mDDPCR could detect MAF as low as 0.01%.^15^, ^25^

The phenomenon of mosaicism was prevalent in neurodevelopmental diseases, such as Dravet syndrome and *PCDH19*‐related epilepsy.^15^, ^26^, ^27^ In the family of A065, two siblings were both diagnosed with AHC carrying the same pathogenic variant c.2401G>A/p.(Asp801Asn), however, this variant was not detected in father's blood. By mDDPCR, MAF of 12.42% was detected in his sperm. When two siblings were affected in one family, parental mosaicism should be considered. Genetic counseling and appropriate prenatal diagnosis are strongly recommended. Mosaicism might have originated after the progenitor cells of the germline tissue diverged from somatic tissues. These results alerted us that using samples from the parental germline might provide more important information compared with peripheral samples in clinical genetic testing. Testing for mosaicism in all available tissues could enable us to determine the stage of occurrence of the deleterious variants. For “*de novo*” pathogenic variants in genetic diseases, more sensitive techniques may be performed to detect unrecognized low levels of parental mosaic variant that may be delivered to the offspring. We also believe that the use of mDDPCR and PASM could help to validate potential mosaicism in families with AHC and other genetic disorders with seemingly recurrent *de novo* variants.^17^

Mosaicism in the AHC proband is also reported for the first time in this study. The patient (A01203) was clinically diagnosed as an atypical AHC case, because his age of onset was later than 18 months. His pathogenic variant was identified as *de novo* after validation in his parents. The variant c.2839G>C/p.(Gly947Arg) was reported in other studies^3^ and was confirmed to be related to the AHC phenotype. In other studies, Panagiotakaki et al reported p.Gly947Arg appeared to correlate with the most favorable prognosis, compared to the other two frequent variants (p.Asp801Asn and p.Glu815Lys).^28^ Our observations were consistent with other studies. In this study, five probands, including A01203, carried the same variant p.G947R. The onset age of A01203 was later, the frequency and duration of hemiplegia were less severe, and his developmental delay was milder, compared with the other four probands. MAF measured from the blood sample in A01203 was 44.19%, which was significantly lower than all the other probands (Figure 3B). In this proband, mosaicism was only tested in the DNA from peripheral blood. We would like to look at levels of mosaicism in other tissue types if the patient consents to participate in the testing. It is reasonable to presume that MAF in the proband's brain might be even lower, contributing to the phenotypic differences. The observation of attenuated phenotype in the mosaic probands is in accordance with our previous report in Dravet syndrome,^26^ however, we still need more patients to study the mosaicism in AHC probands. Mosaicism in the proband has been reported in other genes such as *PCDH19* (8.3%, 6/73), *SCN1A* (1.3%, 4/320), *SCN2A* (6.4%, 7/110), and *CDKL5* (8.8%, 8/91), according to a recent large‐scale study of epilepsy‐related neurodevelopmental disorders.^29^

Based on our observations^15^, ^16^, ^30^ and others' reports,^31^, ^32^, ^33^ detectable mosaic variants are derived either from early embryonic development or from clonal expansion during cell proliferation. Of the six validated parental mosaic cases, four were paternal and two were maternal. More paternal mosaicism than maternal mosaicism was observed. Regarding the four maternal pathogenic variants detected by ASPCR, mDDPCR could not detect mosaicism from the blood of carriers of the maternal pathogenic variants. As female germ cells were not collected and screened for mosaicism, variants that could only be observed in female germline and not in other tissues were likely to be missed in our study, and thus the overall proportion of maternal mosaicism cases might have been underestimated.

We observed that the differences in MAF between tissues showed similarities between mesoderm and ectoderm‐derived samples, similar to our previous observations for samples from Dravet syndrome families.^15^ We also found that mosaic variants that were detectable in paternal blood could be detected in their sperm, although the sample size was small. However, MAFs in the sperm of AHC fathers are not always higher than those in their blood. The PureSperm assay we used to purify male germline cells with reproductive activity could guarantee the vitality of the sperm. Thus, MAFs measured in the sperm samples directly reflected the proportion of sperm carrying deleterious alleles at the time of donation. Our results emphasize the urgency of including sperm detection as well as multiple parental samples for genetic testing and counseling for parents who have already had a child with AHC.

Based on our published results in Dravet syndrome cohorts, probands carrying mosaicism with a higher MAF suggest a disease‐related phenotype.^15^ Six parents with lower level MAF of *ATP1A3* mosaicism (<18.82%) were asymptomatic. One mosaic proband with higher level MAF of *ATP1A3* mosaicism (44.19%) had milder phenotype compared with other heterozygous *ATP1A3* AHC patients. We speculated that MAF of mosaicism was related to the severity of clinical manifestations. Parents carrying lower MAF mosaicism who were missed by panel NGS sequencing, whole‐exome sequencing, or Sanger sequencing tended to be asymptomatic.^15^, ^26^ For the seven probands without *ATP1A3* variant, the more likely hypothesis is that additional pathogenic genes are responsible for AHC. We are planning to carry out whole‐exome sequencing to identify new pathogenic genes.

In conclusion, this is the first report to identify parental mosaicisms of *ATP1A3* in AHC families. In this study, we showed that 7.5% (6/80) of presumed “*de novo*” *ATP1A3* variants with AHC were discovered to be mosaic, and MAF of mosaicism was speculated to be related to severity of clinical manifestations. These findings have significant implications in genetic counseling for AHC patients.

## CONFLICT OF INTEREST

The authors declare no conflict of interest.

### Author contributions

X.Y.1, X.Y.2, Q.W., S.L., Q.Z., and J.C. collected the data. Y.Z., X.Y.1, S.L.,Q.Z., and J.C. performed the clinical diagnoses and recruited the patients. Q.W., X.Y.2, and X.Y.1 designed the experiments. X.Y.2, A.Y.H., and A.Y.Y. carried out the bioinformatics and statistical analyses. X.Y.1, X.Y.2, Y.Z., Q.W., L.W., A.Y.H., and A.Y.Y. wrote and revised the manuscript. Y.Z., L.W., Q.W., X.W., and Y.J. conceived the project.

## Supporting information


**Figure S1.** Genetic screen for mutated *ATP1A3* AHC probands. Sanger sequencing or panel NGS sequencing were carried out using blood samples from 105 AHC probands. A, Among them, 98 probands had pathogenic *ATP1A3* variants. B‐D, The functional effects of the variants were predicted by iFish (B), Polyphen2 trained by HDIV (C), and by HVAR (D). All the* in silico* functional predictions showed that variants from this study had similar deleterious probabilities and were predicated to have more severe functional effects compared with benign missense variants in the *ATP1A3* coding region recorded in ExAC
**Figure S2.** Prediction of variant functions using an unweighted logistic classifier. Benign variants from the1000 Genomes databases and NHLBI Exome Sequencing Project are distinguished from reported AHC causal variants and variants newly reported in this study. The Y axis shows the mean minimal distance from the mutated site to the metal ion binding pocket of the E1 and E2 conformation of the wild‐type protein structure. The X axis shows the number of β‐carbon atoms within 10 Å around the mutated site in the E2 wild‐type protein structure. Different colors show the different types of variants, and the size of each dot shows the number of AHC patients with the *ATP1A3* variant
**Figure S3.** Family A036 is shown here as an index family for a maternally originated allele using allele‐specific PCR (ASPCR) analysis. A, Informative alleles of rs10425063 (chr19:42474864) were used for allele‐of‐origin detection. It is 432 bp from the target genomic position. B, Primers and amplification directions are provided. C‐a, Conventional PCR amplification in the proband indicated the variant position. C‐b, ASPCR amplification in the proband with the primer for the paternal allele indicated that the variant allele was inherited from the father. C‐c, ASPCR amplification in the proband with the maternal allele primer indicated that the wild‐type allele was inherited from the mother
**Figure S4.** PCR Sanger sequencing for Family A065 and the pedigree chart. A, In Family A065, both the proband (II‐1) and his non‐twin sister (II‐2) were affected with AHC. B, The heterozygous variant NM_152296.4:c.2401G>A/NP_689509.1:p.(Asp801Asn) in *ATP1A3* was detected in both patients but in neither of the parents
**Figure S5.** Allele‐specific PCR amplification of the allele of origin for mutant alleles from Family A065. A, Informative alleles of rs10425063 (chr19:42474864) were used for allele‐of‐origin detection. It is 517 bp from the target genomic position. B, Primers and amplification directions are provided. C‐a, Conventional PCR amplification in the proband showed the variant position. C‐b, ASPCR amplification in the proband with the primer for the paternal allele indicated that the variant allele was inherited from the father. C‐c, ASPCR amplification in the proband with the maternal allele primer indicated the wild‐type allele was inherited from the mother. D‐a, Conventional PCR amplification in the sister of the proband showed the variant position. D‐b, ASPCR amplification in the sister of the proband with the primer for the paternal allele indicated that the variant allele originated from the father. D‐c, ASPCR amplification in the sister of the proband with the primer for the maternal allele indicated that the wild‐type allele was from the mother
**Figure S6.** Endpoint genotyping qPCR analysis for TaqMan MGB assays designed for *ATP1A3* variants. Customized TaqMan MGB genotyping assays were designed under part number 4331349. IDs for assays are provided along with the base substitutions and amino acid substitutions. All variants are based on the reference cDNA sequence NM_152296.4 and amino acid sequence NP_689509.1. The endpoint genotyping reactions were carried out on a StepOne Plus real‐time PCR system. Peripheral blood DNA samples from the putative heterozygous proband, their parents, as well as healthy control and non‐template control (NTC) samples were detected. The samples are separated by color and shape on the allele discrimination plot. Heterozygous samples with both alleles are shown at the top right corner of the plot. Samples showing largely the wild‐type allele are shown at the bottom right corner of the plot. NTCs are supposed to appear at the bottom left corner. Owing to different DNA concentrations and assay performances, signals at the bottom sometimes spread across the X axis
**Figure S7.** Determination of the binomial distribution cutoff of MAF in mDDPCR according to the negative control samples. TaqMan assays were tested using blood from normal controls to identify the cutoff for the reference homozygous signals. The empirical error rate of the assays was estimated to be 2.15664e‐05 according to the estimation of total droplets detected as wild‐type and mutant from all the negative control mDDPCR results. The dashed black line shows the upper bound of the 95% binominal CIs under different numbers of droplets containing the target sequence. According to the cutoff, an MAF of 0.01% was set as the cutoff for the 95% binominal CI detected by mDDPCR; the cutoff is shown as the horizontal dashed blue line. The cutoff was above the theoretical value for a number of droplets larger than 10 000, which is shown as a vertical dashed gray line
**Figure S8.** Flow cytometry scatter plots for all candidate parental mosaic families. PMT1 and PMT2 are the raw signals from the photomultiplier tubes. WT and MU denote droplets containing wild‐type and mutant genomic sequences. NA denotes droplets containing non‐target genomic sequences. Numbers under each cluster denote the percentage of the droplet above all detected droplets. All collected and detected sample are provided. In positive mosaic samples, a mutant cluster arises in the MU region and the number of MU droplets
**Figure S9.** Distribution of MAF for all families who donated multiple peripheral samples. Multiple samples were collected from 13 parents out of 11 families. Multiple samples include buccal swab (labeled buccal), hair follicles (labeled hair), saliva, skin biopsy (labeled skin), paternal purified sperm (labeled sperm) and urine. MAF from these samples were quantified by mDDPCR, few DNA copies were obtained from the urine sample of A11203 and the variation was large
**Figure S10.** Prenatal diagnosis for Family A067. A, pedigree diagram of A067. The proband (II‐1) was detected with the NM_152296.4:c.2839G>A/NP_689509.1:p.(Gly947Arg) heterozygous variant by Sanger sequencing, PASM, and mDDPCR. The mother (I‐1) was a validated mosaic carrier by PASM and mDDPCR; the MAF of the *ATP1A3* variant in the blood of the mother was 7.65%. B, Sanger sequencing results for the DNA segment (reverse strand) of Family A06701; for I‐1, I‐2, and II‐1, blood samples were detected; for I‐II, DNA from amniocentesis was detected. Variant signals were not observed for I‐2 or II‐2. C, STR analysis for Family A067. Results from markers DXS6797 and DXS6807 are shown
**Table S1.** Gene list of next‐generation sequencing panel
**Table S2.** Primers used for amplification and sequencing of *ATP1A3* for SNP genotyping
**Table S3.** Primers used for allele‐specific amplification of the identified *ATP1A3* polymorphisms
**Table S4.** Primers used for short tandem repeat (STR) analysis for paternity
**Table S5.** Primers used for PASM detection
**Table S6.** Summary for screening of variants in *ATP1A3*
**Table S7.** Parent‐of‐origin analysis by allele‐specific PCR
**Table S8.** Summary for patient variant information

## Data Availability

We have deposited all the variation and phenotype data in the LOVD database (Patient ID 00155754‐00155762, 00163007‐00163023, 00163692‐00163741, 00163984, 00163999‐00164008; variant ID 359660‐359685, 367799‐367848, 368415‐368457, 368462, 368479‐368488). Raw data containing mDDPCR flow cytometry signals (FCS files) are available at https://pan.baidu.com/s/1i5vi5Op under access code “wgt6”.
