# Supplementary material for: ATP1A3 mosaicism in families with alternating hemiplegia of childhood
Source: Clin Genet. 2019 Apr 3;96(1):43–52. doi: 10.1111/cge.13539 (PMC6850116; doi:10.1111/cge.13539)
Supplement: Supplementary file 1 — Figure S1. Genetic screen for mutated ATP1A3 AHC probands. Sanger sequencing or panel NGS sequencing were carried out using blood samples from 105 AHC probands. A, Among them, 98 probands had pathogenic ATP1A3 variants. B‐D, The functional effects of the variants were predicted by iFish (B), Polyphen2 trained by HDIV (C), and by HVAR (D). All the in silico functional predictions showed that variants from this study had similar deleterious probabilities and were predicated to have more severe functional effects compared with benign missense variants in the ATP1A3 coding region recorded in ExAC Figure S2. Prediction of variant functions using an unweighted logistic classifier. Benign variants from the1000 Genomes databases and NHLBI Exome Sequencing Project are distinguished from reported AHC causal variants and variants newly reported in this study. The Y axis shows the mean minimal distance from the mutated site to the metal ion binding pocket of the E1 and E2 conformation of the wild‐type protein structure. The X axis shows the number of β‐carbon atoms within 10 Å around the mutated site in the E2 wild‐type protein structure. Different colors show the different types of variants, and the size of each dot shows the number of AHC patients with the ATP1A3 variant Figure S3. Family A036 is shown here as an index family for a maternally originated allele using allele‐specific PCR (ASPCR) analysis. A, Informative alleles of rs10425063 (chr19:42474864) were used for allele‐of‐origin detection. It is 432 bp from the target genomic position. B, Primers and amplification directions are provided. C‐a, Conventional PCR amplification in the proband indicated the variant position. C‐b, ASPCR amplification in the proband with the primer for the paternal allele indicated that the variant allele was inherited from the father. C‐c, ASPCR amplification in the proband with the maternal allele primer indicated that the wild‐type allele was inherited from the mother Figure S4. PCR Sange [file CGE-96-43-s001.docx]

**Supplementary information**


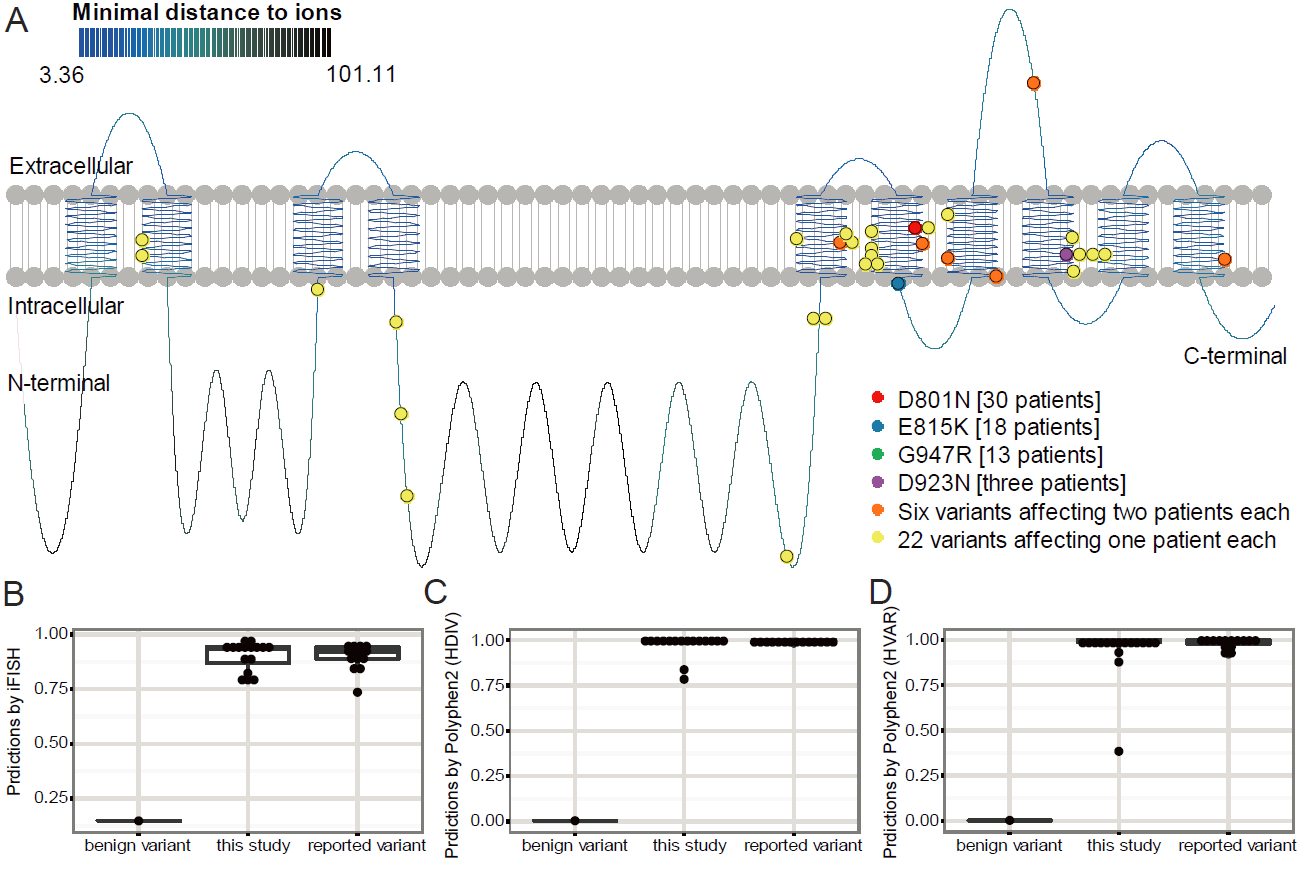


**Figure S1.** **Genetic screen for mutated *ATP1A3* AHC probands.** Sanger sequencing or panel NGS sequencing were carried out using blood samples from 105 AHC probands. A: Among them, 98 probands had pathogenic *ATP1A3* variants. B-D: The functional effects of the variants were predicted by iFish (B), Polyphen2 trained by HDIV (C), and by HVAR (D). All the *insilico* functional predictions showed that variants from this study had similar deleterious probabilities and were predicated to have more severe functional effects compared with benign missense variants in the *ATP1A3* coding region recorded in ExAC.


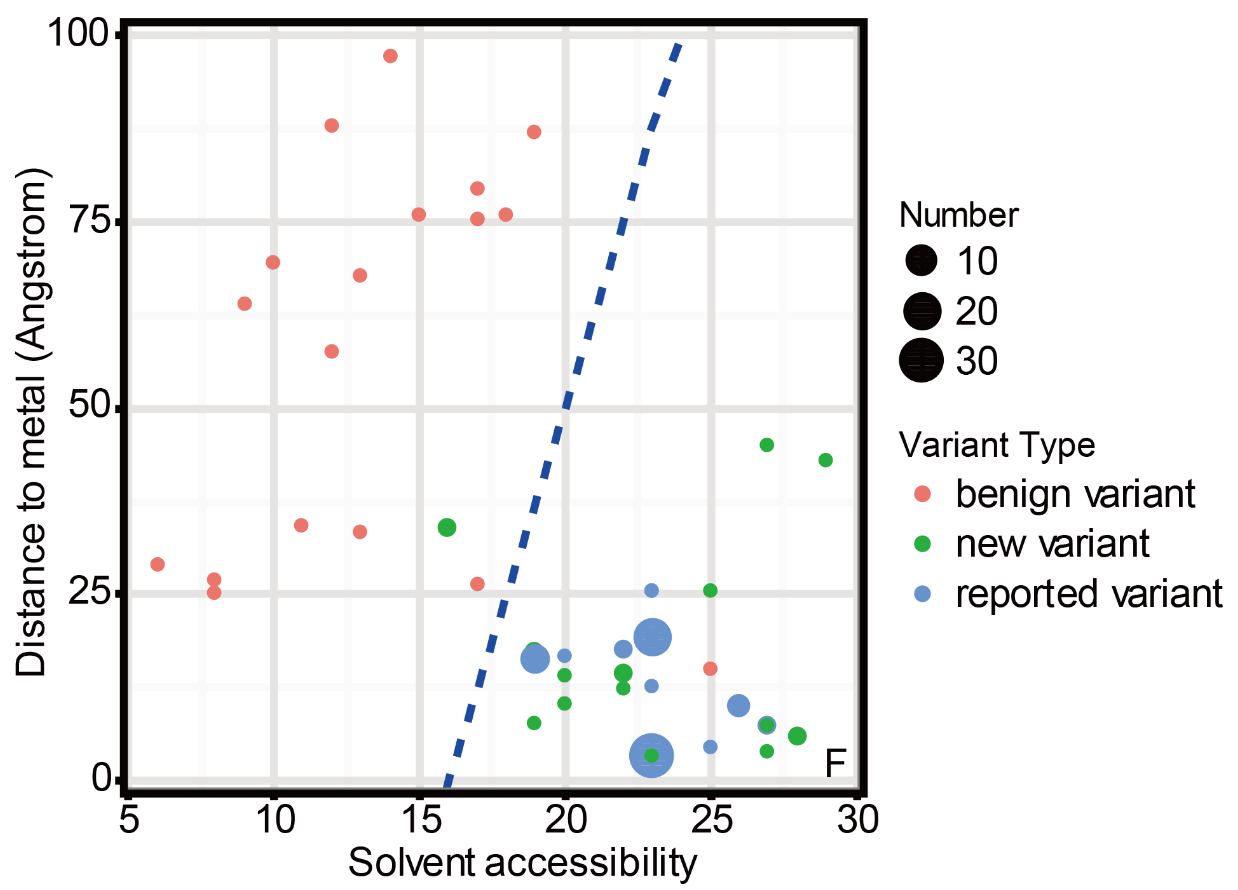


**Figure S2: Prediction of variant functions using an unweighted logistic classifier.** Benign variants from the1000 Genomes databases and NHLBI Exome Sequencing Project are distinguished from reported AHC causal variants and variants newly reported in this study. The Y axis shows the mean minimal distance from the mutated site to the metal ion binding pocket of the E1 and E2 conformation of the wild-type protein structure. The X axis shows the number of β-carbon atoms within 10 Å around the mutated site in the E2 wild-type protein structure. Different colors show the different types of variants, and the size of each dot shows the number of AHC patients with the *ATP1A3* variant.


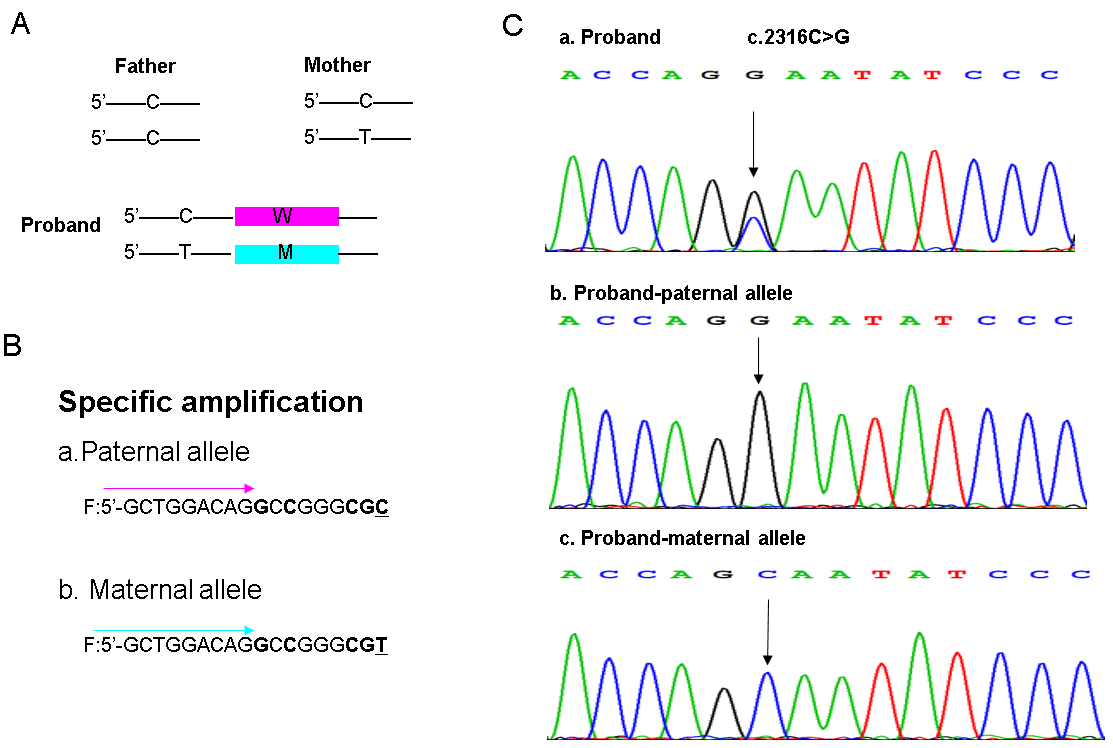


**Figure S3. Family A036 is shown here as an index family for a maternally originated allele using allele-specific PCR analysis**. A: Informative alleles of rs10425063 (chr19:42474864) were used for allele-of-origin detection. It is 432 bp from the target genomic position. B: Primers and amplification directions are provided. C-a: Conventional PCR amplification in the proband indicated the variant position. C-b: ASPCR amplification in the proband with the primer for the paternal allele indicated that the variant allele was inherited from the father. C-c: ASPCR amplification in the proband with the maternal allele primer indicated that the wildtype allele was inherited from the mother.


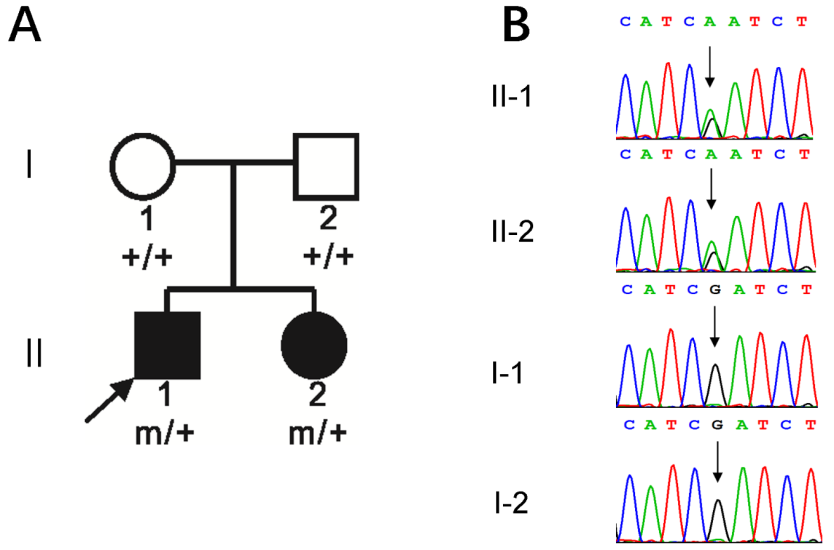


**Figure S4. PCR Sanger sequencing for Family A065 and the pedigree chart.** A: In Family A065, both the proband (II - 1) and his non-twin sister (II - 2) were affected with AHC. B: The heterozygous variant NM_152296.4:c.2401G>A/NP_689509.1:p.(Asp801Asn) in *ATP1A3*was detected in both patients but in neither of the parents.


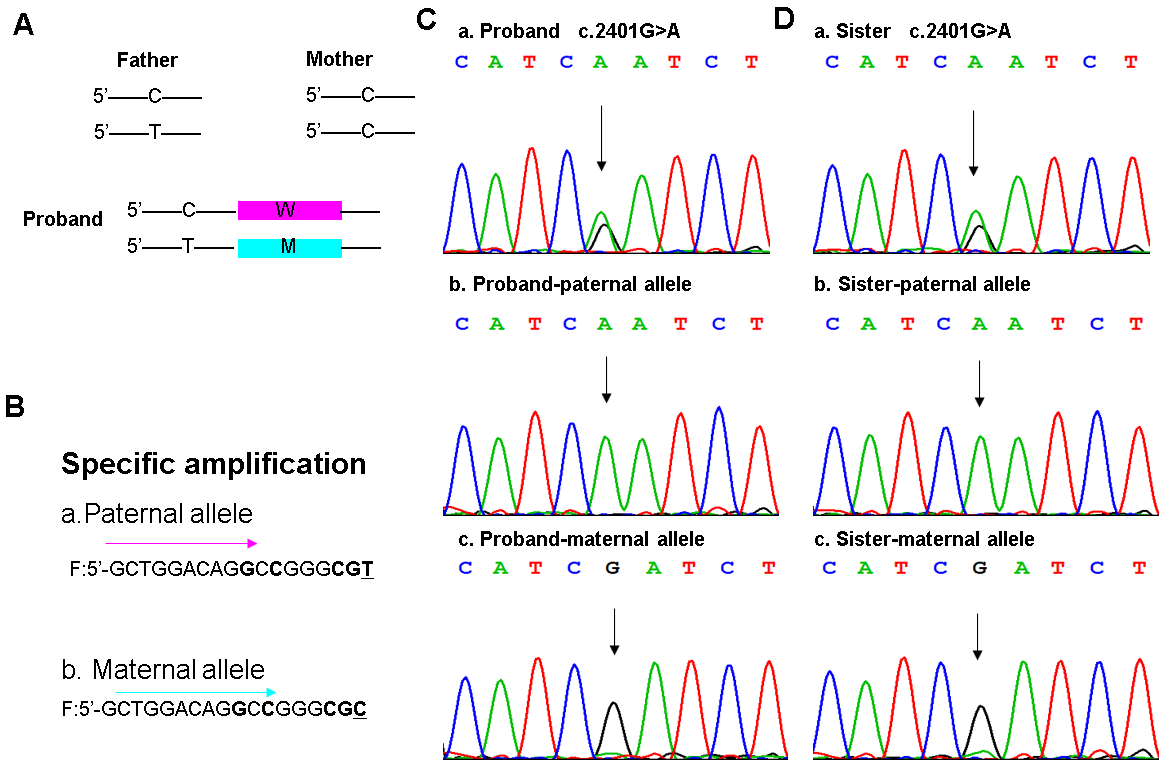


**Figure S5. Allele-specific PCR amplification of the allele of origin for mutant alleles from Family A065.** A: Informative alleles of rs10425063 (chr19:42474864) were used for allele-of-origin detection. It is 517 bp from the target genomic position. B: Primers and amplification directions are provided. C-a: Conventional PCR amplification in the proband demonstrated the variant position. C-b: ASPCR amplification in the proband with the primer for the paternal allele indicated that the variant allele was inherited from the father. C-c: ASPCR amplification in the proband with the maternal allele primer indicated the wildtype allele was inherited from the mother. D-a: Conventional PCR amplification in the sister of the proband demonstrated the variant position. D-b: ASPCR amplification in the sister of the proband with the primer for the paternal allele indicated that the variant allele originated from the father. D-c: ASPCR amplification in the sister of the proband with the primer for the maternal allele indicated that the wildtype allele was from the mother.

| ●Proband●Father●Mother**×**Healthy Control ∎Non Template Control(NTC) | |
| --- | --- |
| 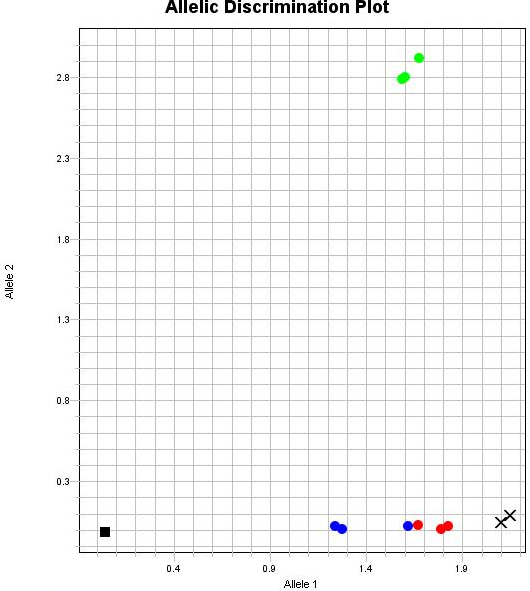 | Assay ID  AH0JGHG  Amino acid substitution  NP_689509.1:p.(Thr133Pro)  Base substitution  NM_152296.4:c.397A>C  Related Families  A092 |
| 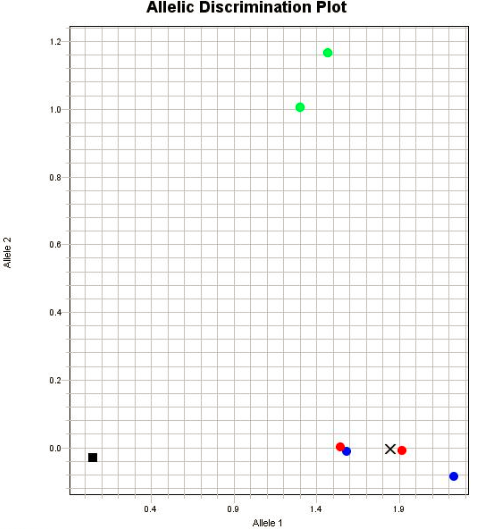 | Assay ID  AH1SDTP  Amino acid substitution  NP_689509.1:p.(Ser137Tyr)  Base substitution  NM_152296.4:c.410C>A  Related Families  A021 |
| 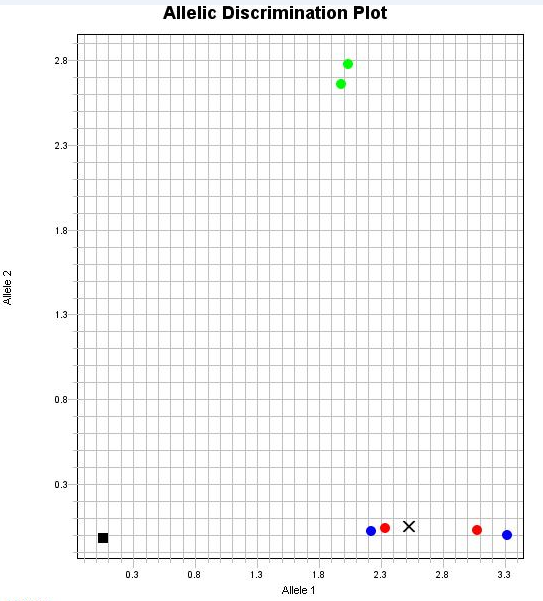 | Assay ID  AH21BZX  Amino acid substitution  NP_689509.1:p.(Glu277Lys)  Base substitution  NM_152296.4:c.829G>A  Related Families  A081 |
| 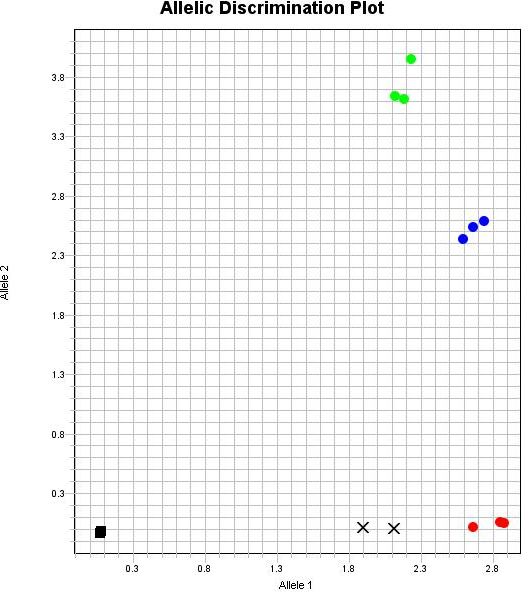 | Assay ID  AHUAP18  Amino acid substitution  NP_689509.1:p.(Glu355del)  Base substitution  NM_152296.4:c.1063_1065del  Related Families  A112 |
| 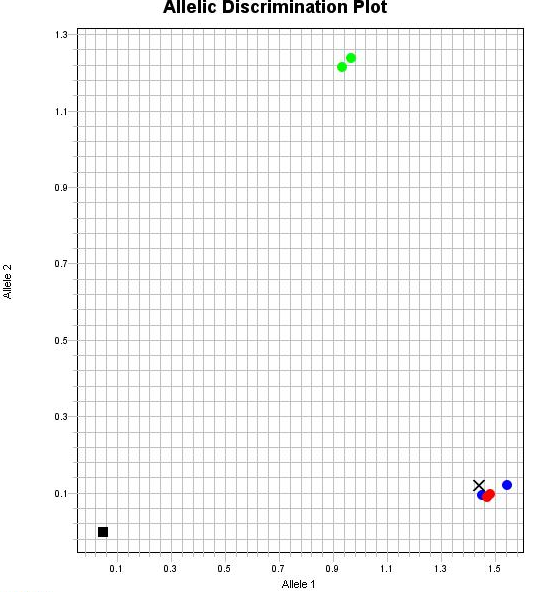 | Assay ID  AH39955  Amino acid substitution  NP_689509.1:p.(Gly706Arg)  Base substitution  NM_152296.4:c.2116G>A  Related Families  A050 |
| 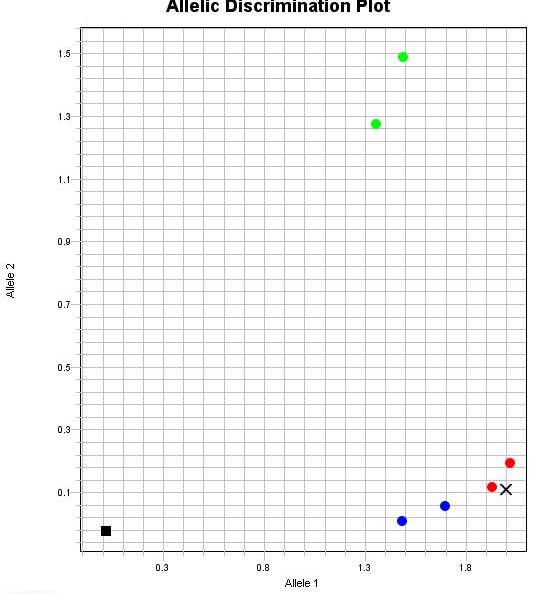 | Assay ID  AH5I8CD  Amino acid substitution  NP_689509.1:p.(Gly755Ser)  Base substitution  NM_152296.4:c.2263G>A  Related Families  A032 |
| 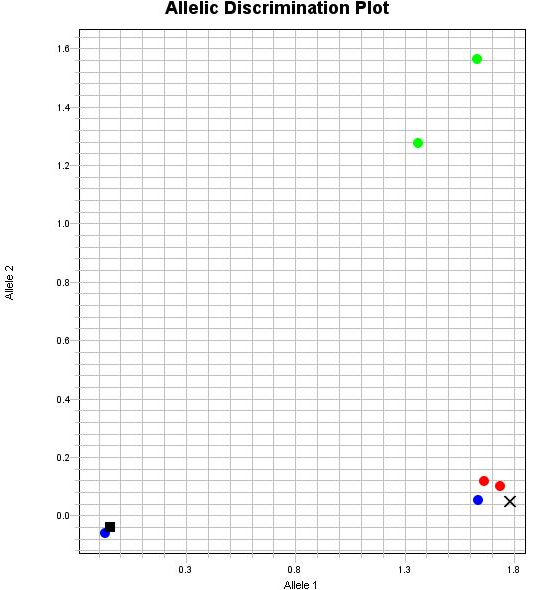 | Assay ID  AH6R6IL  Amino acid substitution  NP_689509.1:p.(Gly755Cys)  Base substitution  NM_152296.4:c.2263G>T  Related Families  A064 |
| 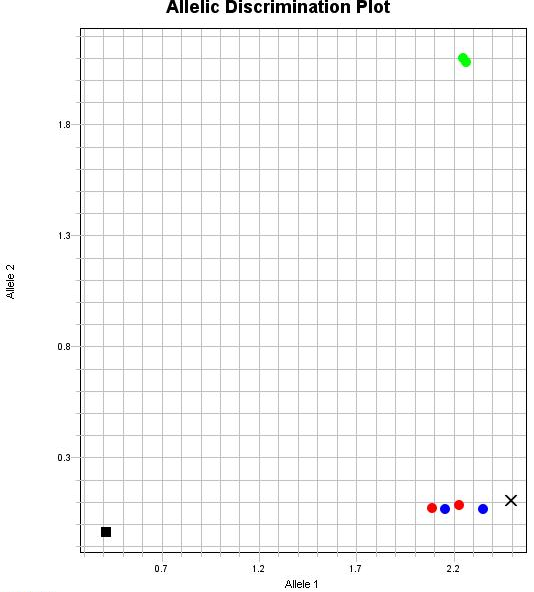 | Assay ID  AH704OT  Amino acid substitution  NP_689509.1:p.(Thr771Asn)  Base substitution  NM_152296.4:c.2312C>A  Related Families  A026, A108 |
| 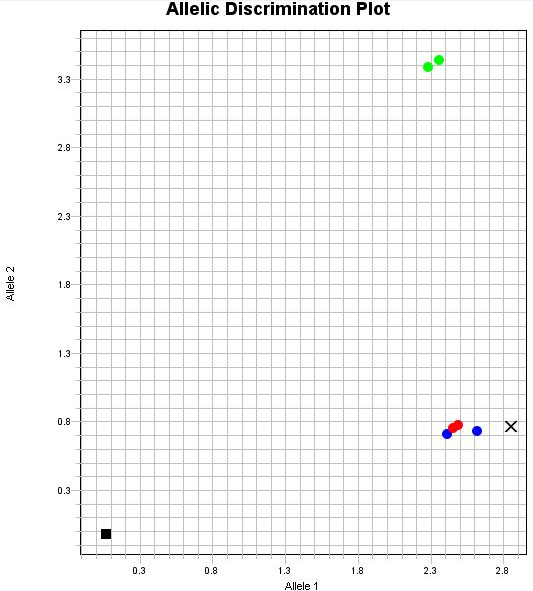 | Assay ID  AH892U1  Amino acid substitution  NP_689509.1:p.(Ser772Arg)  Base substitution  NM_152296.4:c.2316C>G  Related Families  A036 |
| 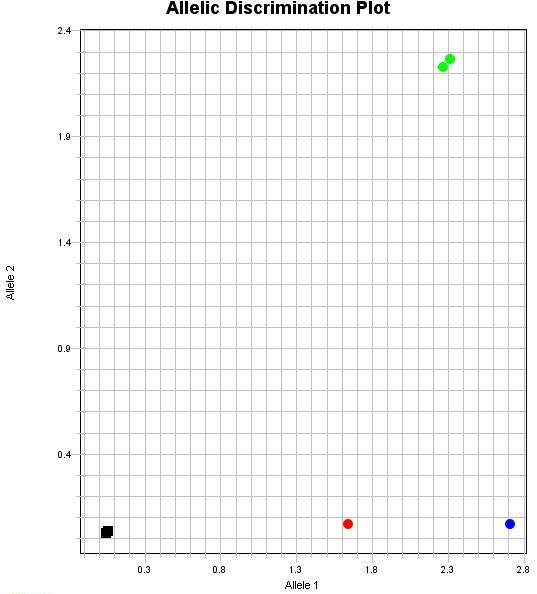 | Assay ID  AH1SCW1  Amino acid substitution  NP_689509.1:p.(Asp801Asn)  Base substitution  NM_152296.4:c.2401G>A  Related Families  A011, A020, A022, A024, A025, A028, A031, A035, A037, A039, A042, A046, A048, A061, A065, A080, A086, A087, A089, A091, A093, A095, A097, A100, A111 |
| 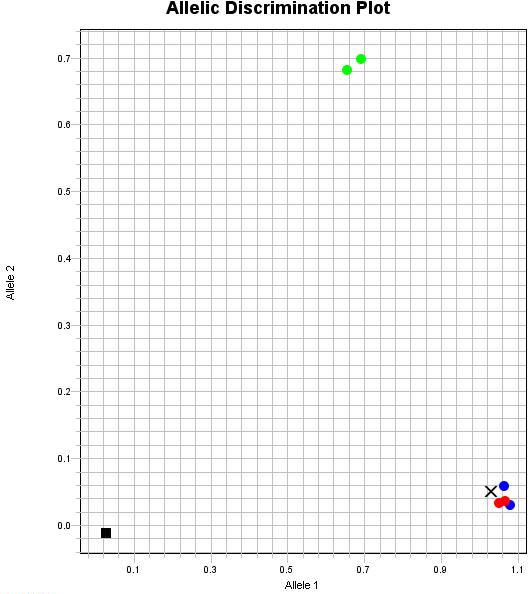 | Assay ID  AHUAPR7  Amino acid substitution  NP_689509.1:p.(Asp801Glu)  Base substitution  NM_152296.4:c.2403T>A  Related Families  A107 |
| 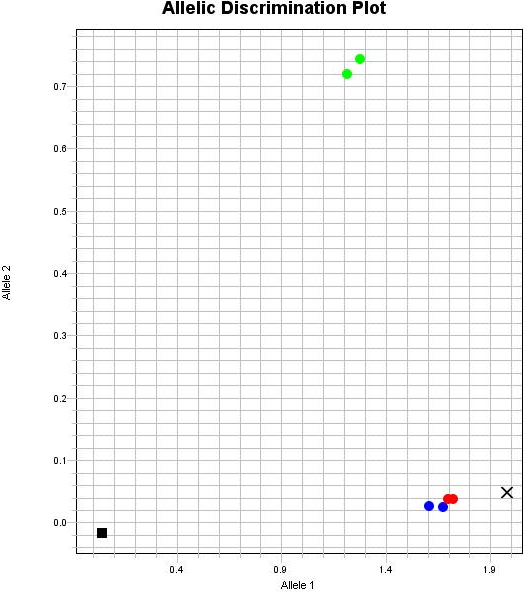 | Assay ID  AHABH0U  Amino acid substitution  NP_689509.1:p.(Leu802Pro)  Base substitution  NM_152296.4:c.2405T>C  Related Families  A056 |
| 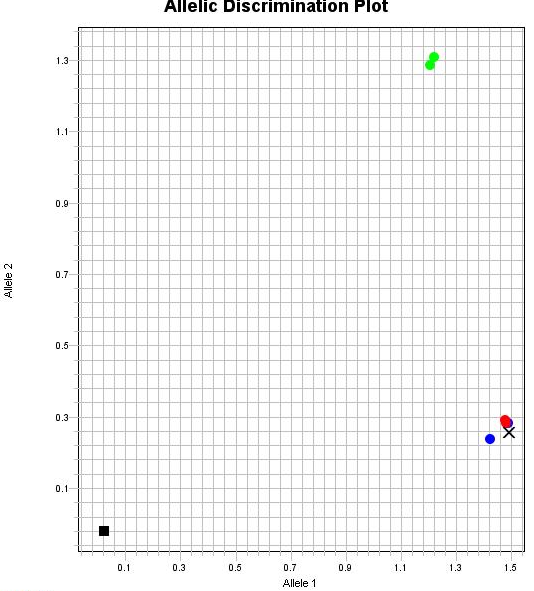 | Assay ID  AHBKF62  Amino acid substitution  NP_689509.1:p.(Asp805His)  Base substitution  NM_152296.4:c.2413G>C  Related Families  A062, A098 |
| 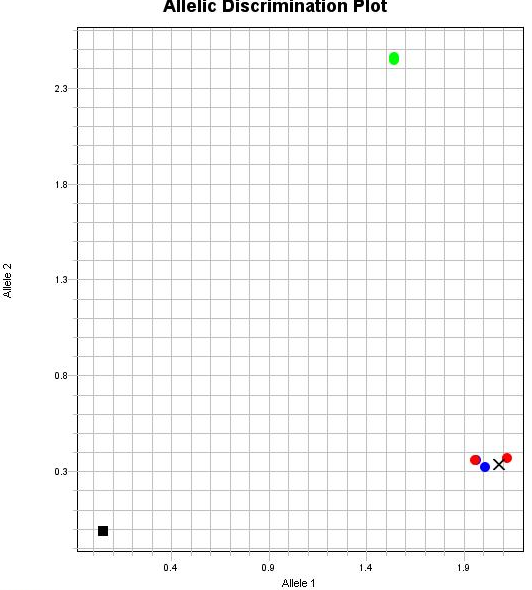 | Assay ID  AHCTEDA  Amino acid substitution  NP_689509.1:p.(Pro808Leu)  Base substitution  NM_152296.4:c.2423C>T  Related Families  A054 |
| 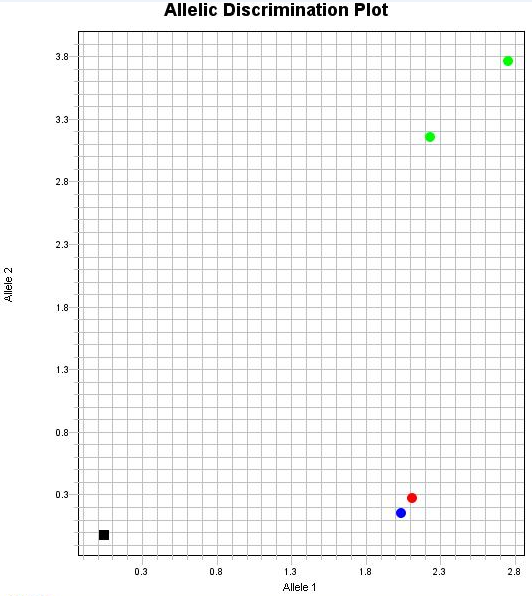 | Assay ID  AH21A29  Amino acid substitution  NP_689509.1:p.(Glu815Lys)  Base substitution  NM_152296.4:c.2443G>A  Related Families  A005, A007, A013, A015, A023, A033, A040, A060, A063, A073, A074, A079, A094, A101, A102, A105, A106 |
| 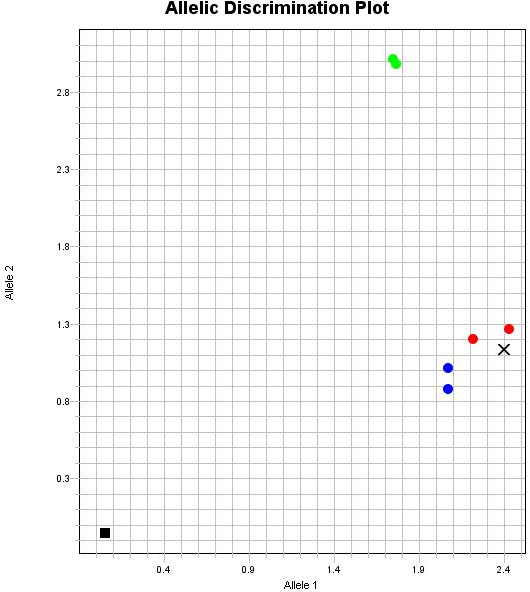 | Assay ID  AHD2CJI  Amino acid substitution  NP_689509.1:p.(Leu834Ser)  Base substitution  NM_152296.4:c.2501T>C  Related Families  A070,A077 |
| 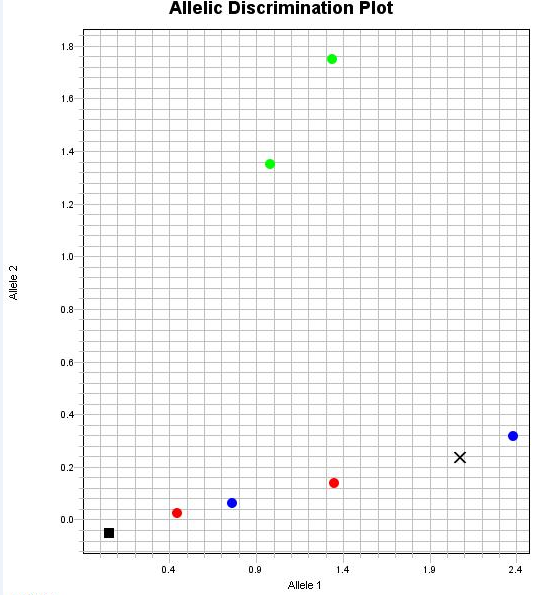 | Assay ID  AHFBAPQ  Amino acid substitution  NP_689509.1:p.(Leu839Pro)  Base substitution  NM_152296.4:c.2516T>C  Related Families  A059, A076 |
| 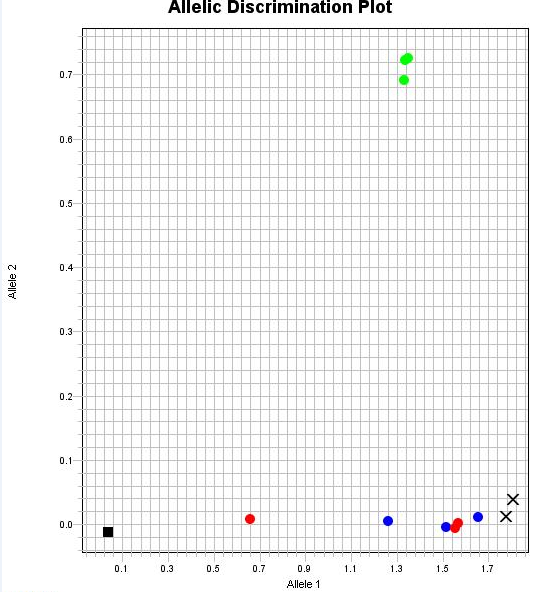 | Assay ID  AHGJ8VY  Amino acid substitution  NP_689509.1:p.(Gln851Pro)  Base substitution  NM_152296.4:c.2552A>C  Related Families  A069 |
| 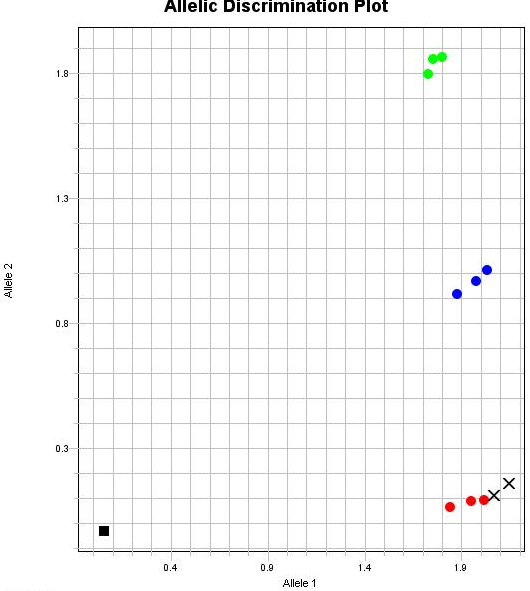 | Assay ID  AHHS616  Amino acid substitution  NP_689509.1:p.(Gly893Arg)  Base substitution  NM_152296.4:c.2677G>A  Related Families  A052, A071 |
| 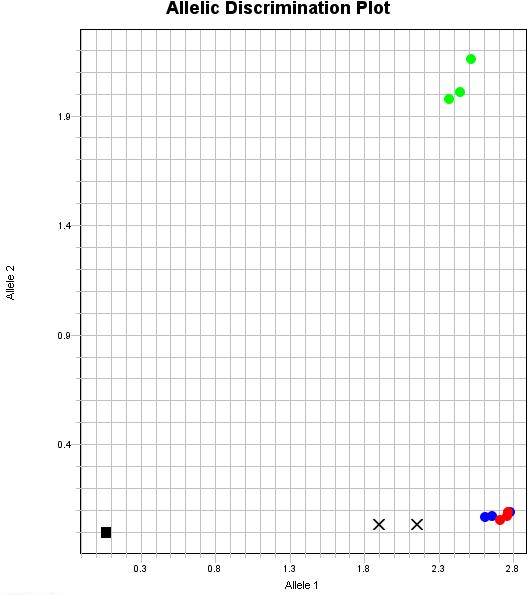 | Assay ID  AHWSMF9  Amino acid substitution  NP_689509.1:p.(Val919del)  Base substitution  NM_152296.4:c.2755_2757del  Related Families  A113 |
| 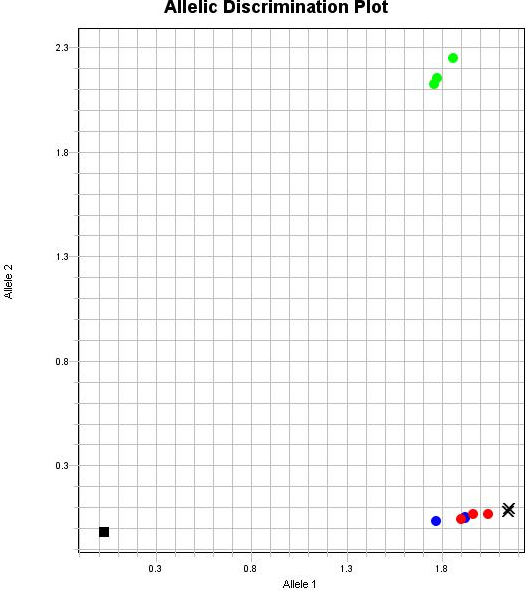 | Assay ID  AHI148E  Amino acid substitution  NP_689509.1:p.(Asp923Asn)  Base substitution  NM_152296.4:c.2767G>A  Related Families  A041, A075 |
| 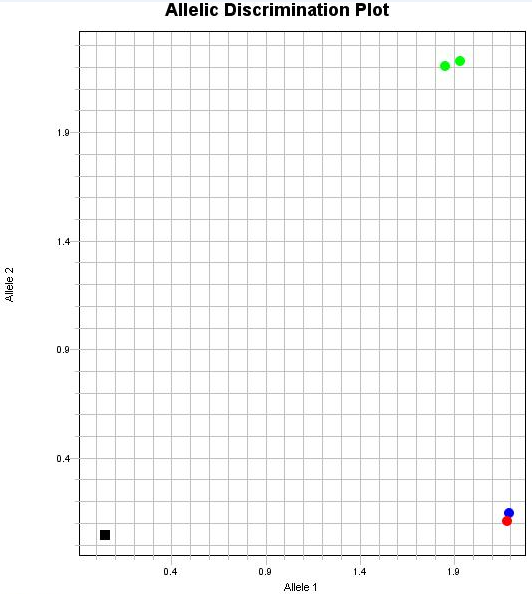 | Assay ID  AH3989H  Amino acid substitution  NP_689509.1:p.(Gly947Arg)  Base substitution  NM_152296.4:c.2839G>A  Related Families  A001, A002, A016, A030, A053, A067, A085, A096 |
| 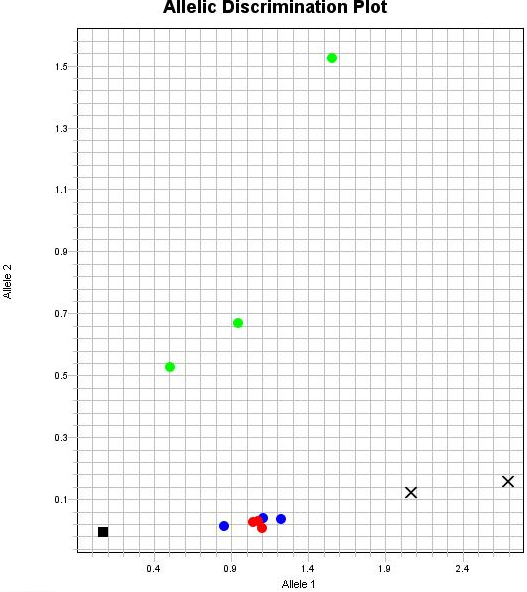 | Assay ID  AHMSZQ2  Amino acid substitution  NP_689509.1:p.(Gly947Arg)  Base substitution  NM_152296.4:c.2839G>C  Related Families  A012, A055, A066, A082 |
| 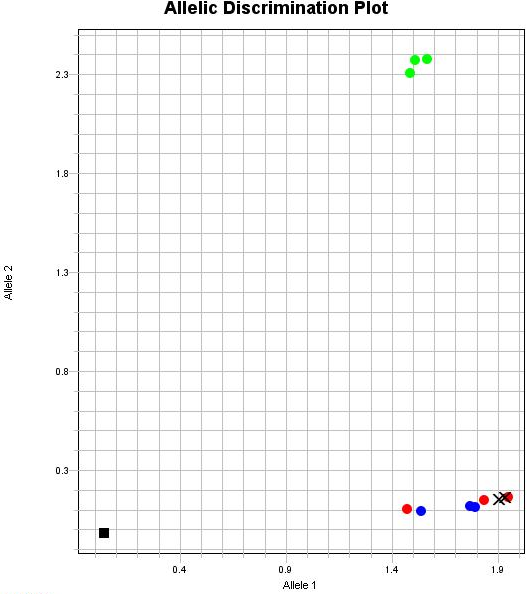 | Assay ID  AHN1XXA  Amino acid substitution  NP_689509.1:p.(Asp992Tyr)  Base substitution  NM_152296.4:c.2974G>T  Related Families  A072 |

**Figure S6.** **Endpoint genotyping qPCR analysis for TaqMan MGB assays designed for *ATP1A3* variants.** Customized TaqMan MGB genotyping assays were designed under part number 4331349. IDs for assays are provided along with the base substitutions and amino acid substitutions. All variants are based on the reference cDNA sequence NM_152296.4 and amino acid sequence NP_689509.1. The endpoint genotyping reactions were carried out on a Step One Plus real-time PCR system. Peripheral blood DNA samples from the putative heterozygous proband, their parents, as well as healthy control and non-template control (NTC) samples were detected. The samples are separated by color and shape on the allele discrimination plot. Heterozygous samples with both alleles are shown at the top right corner of the plot. Samples showing largely the wild-type allele are shown at the bottom right corner of the plot. NTCs are supposed to appear at the bottom left corner. Owing to different DNA concentrations and assay performances, signals at the bottom sometimes spread across the X axis.


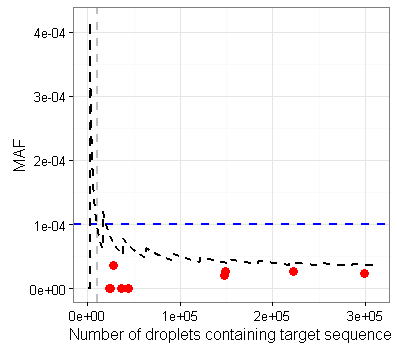


**Figure S7. Determination of the binomial distribution cutoff of MAF in mDDPCR according to the negative control samples.** TaqMan assays were tested using blood from normal controls to identify the cutoff for the reference homozygous signals. The empirical error rate of the assays was estimated to be 2.15664e-05 according to the estimation of total droplets detected as wildtype and mutant from all the negative control mDDPCR results. The dashed black line shows the upper bound of the 95% binominal CIs under different numbers of droplets containing the target sequence. According to the cutoff, an MAF of 0.01% was set as the cutoff for the 95% binominal CI detected by mDDPCR; the cutoff is shown as the horizontal dashed blue line. The cutoff was above the theoretical value for a number of droplets larger than 10000, which is shown as a vertical dashed gray line.


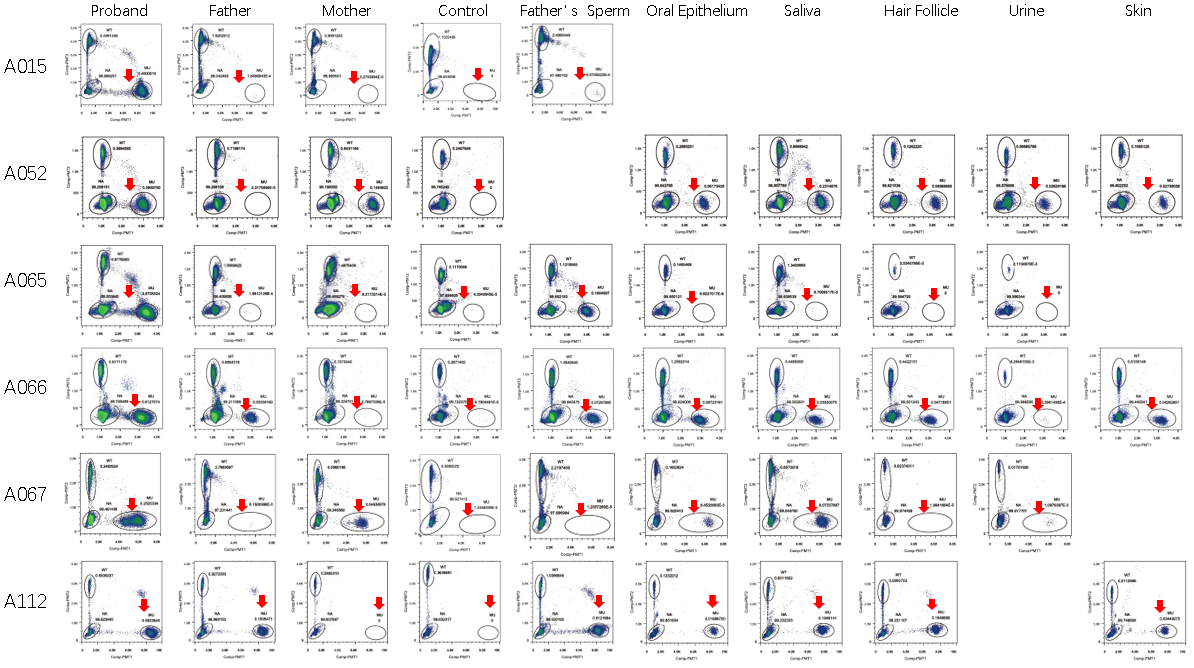


**Figure S8. Flow cytometry scatter plots for all candidate parental mosaic families. PMT1 and PMT2 are the raw signals from the photomultiplier tubes.** WT and MU denote droplets containing wild-type and mutant genomic sequences. NA denotes droplets containing non-target genomic sequences. Numbers under each cluster denote the percentage of the droplet above all detected droplets. All collected and detected sample are provided. In positive mosaic samples, a mutant cluster arises in the MU region and the number of MU droplets.


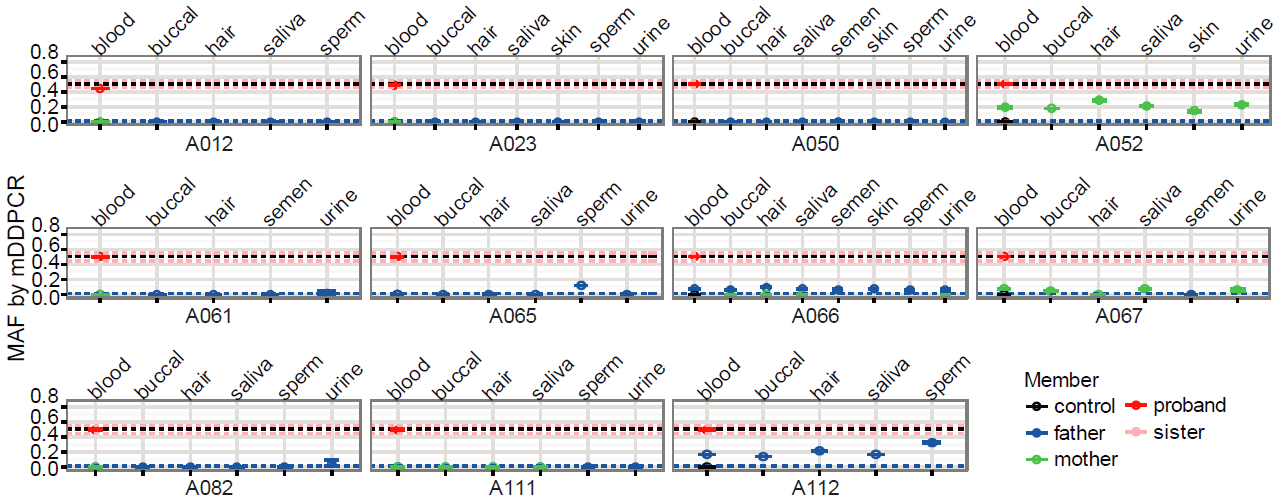


**Figure S9. Distribution of MAF for all families who donated multiple peripheral samples.** Multiple samples were collected from thirteen parents out of eleven families. Multiple samples include buccal swab (labeled buccal), hair follicles (labeled hair), saliva, skin biopsy (labeled skin), paternal purified sperm (labeled sperm) and urine. MAF from these samples were quantified by mDDPCR, few DNA copies were obtained from the urine sample of A11203 and the variation was large.


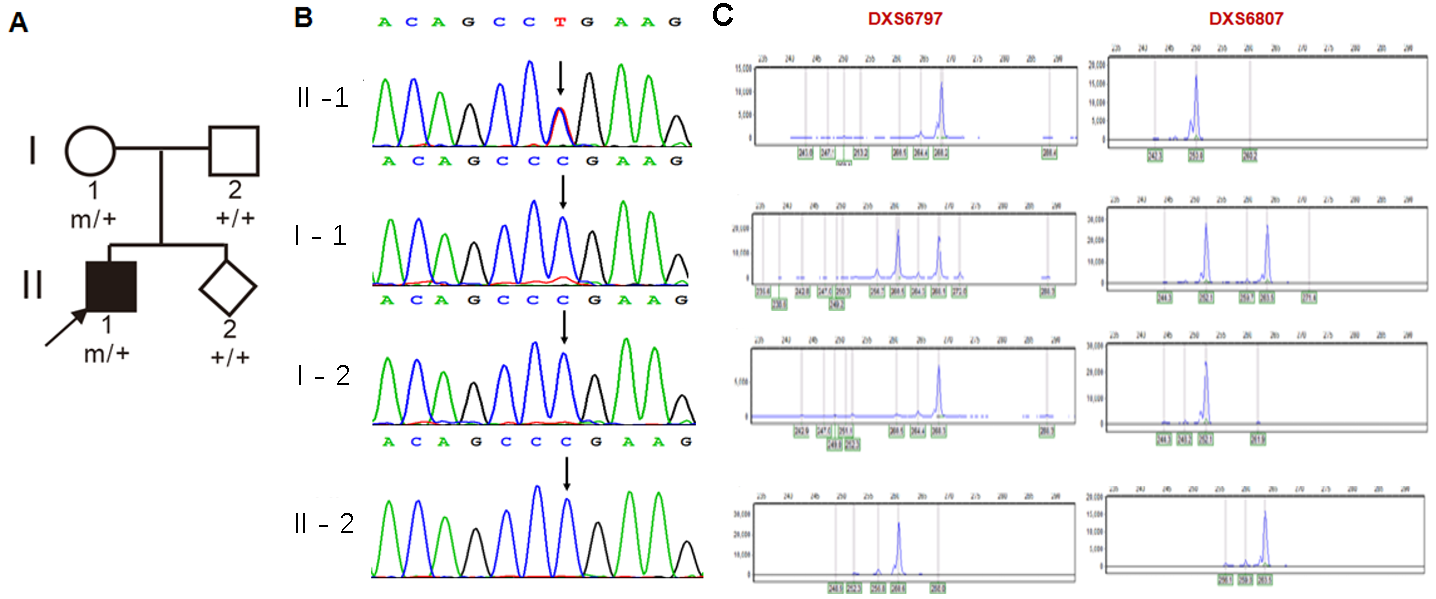


**Figure S10.** **Prenatal diagnosis for Family A067.** A: pedigree diagram of A067. The proband (II-1) was detected with the NM_152296.4:c.2839G>A/ NP_689509.1:p.(Gly947Arg) heterozygous variant by Sanger sequencing, PASM, and mDDPCR. The mother (I-1) was a validated mosaic carrier by PASM and mDDPCR; the MAF of the*ATP1A3*variant in the blood of the mother was 7.65%. B: Sanger sequencing results for the DNA segment (reverse strand) of Family A06701; for I-1, I-2, and II-1, blood samples were detected; for I-II, DNA from amniocentesis was detected. Variant signals were not observed for I-2 or II-2. C: STR analysis for Family A067.Results from markers DXS6797 and DXS6807 are shown.

**Table S1. Gene list of next-generation sequencing panel.**

| ADSL | CHD2 | DHFR | GLB1 | MAGI2 | PNPO | SLC9A6 |
| --- | --- | --- | --- | --- | --- | --- |
| ALDH7A1 | CHRNA2 | DIAPH3 | GLRA1 | MAPK10 | POLG | SPTAN1 |
| ALG13 | CHRNA4 | DNAJC6 | GPR56 | MBD5 | PPT1 | SRPX2 |
| ARG1 | CHRNA7 | DNM1 | GPR98 | MDGA2 | PROC | ST3GAL2 |
| ARHGEF15 | CHRNB2 | DOCK7 | GRIN1 | ME2 | PRRT2 | ST3GAL5 |
| ARHGEF9 | CLCN2 | EEF1A2 | GRIN2A | MECP2 | RBFOX1 | STRADA |
| ARX | CLCN4 | EFHC1 | GRIN2B | MEF2C | RBFOX2 | STXBP1 |
| ASAH1 | CLN3 | ELP4 | HAX1 | MFSD8 | RBFOX3 | SYNGAP1 |
| ATP13A4 | CLN5 | EPHB2 | HDAC4 | MTHFR | RELN | SYNJ1 |
| ATP1A2 | CLN6 | ErbB4 | HEXA | MTOR | RYR3 | SZT2 |
| ATP1A3 | CLN8 | FASN | HEXB | NDE1 | SCN1A | TBC1D24 |
| ATP6AP2 | CNTN5 | FLNA | HNRNPH1 | NEDD4L | SCN1B | TCF4 |
| ATP7A | CNTNAP2 | FOLR1 | HNRNPU | NID2 | SCN2A | TNK2 |
| BRAF | COX6B1 | FOXG1 | IQSEC2 | NRXN1 | SCN8A | TPP1 |
| BSN | CSTB | FOXP2 | KCNB1 | PAFAH1B1 | SHANK3 | TSC1 |
| CACNA1A | CTNNA3 | GABBR2 | KCNH5 | PCDH19 | SLC13A5 | TSC2 |
| CACNA1H | CTSD | GABRA1 | KCNMA1 | PDHA1 | SLC19A3 | TUBA1A |
| CACNB4 | CYB5R3 | GABRA6 | KCNQ2 | PIGA | SLC1A3 | UBE3A |
| CASK | DBH | GABRB2 | KCNQ3 | PIGV | SLC25A22 | VRK2 |
| CDH13 | DCX | GABRB3 | KCNT1 | PLCB1 | SLC2A1 | WDR45 |
| CDH9 | DEPDC5 | GABRD | LGI1 | PNKD | SLC35A2 | ZEB2 |
| CDKL5 | DGKD | GABRG2 | LIAS | PNKP | SLC46A1 |  |

**Table S2**. **Primers used for amplification and sequencing of *ATP1A3* for SNP genotyping.**

| Exon | Primer (5’→3’) | Product(bp) |
| --- | --- | --- |
| 5 | F: CAGACCCAGCCTCTAAAAAAACAC  R: AGCCCATACACTAACACCCCCG | 872 |
| 9 | F: AGCCTGGACGACAGAGCAAGACT  R: CTCAGCCACTTTCTTGTTGCGTT | 1594 |
| 16 | F: GCCATCGCTCTCTCTGCTCTTCC  R: AATCCACCAAGGCATGAACCGT | 1952 |
| 17 | F: GCCAGGTGTGGAGACTTATGCC  R: GGAGGTGGAGAAGGATGGGGT | 1241 |
| 18 | F: GCCAGGTGTGGAGACTTATGCC  R: GGAGGTGGAGAAGGATGGGGT | 1241 |
| 20 | F: CTTCCTACGATGGCCCCCTCAGTC  R: CGTGGTGGGAGCAGCCTATGG | 1875 |
| 21 | F: GGGTTCAAGCGATTCTCATGC  R: TGAGTTTGCGGATTTCGTCGTAG | 1164 |

**Table S3. Primers used for allele-specific amplification of the identified *ATP1A3* polymorphisms.**

| Variant | SNP ID | SNP Genomic Position (hg19) | Primer (5’→3’) | Product (bp) |
| --- | --- | --- | --- | --- |
| 1.T133P | rs2217342 | Chr19:42489516 A/C | F: AGAGGGACTCAAGCACAAACAGG  R: CCGAGTCTCCAAGGGGTTGTCGTGA | 1073 |
| 2. D801N | rs10425063 | Chr19:42474864 G/A | F: AACCCGGGAGGCGGAGCT  R: TCTGCTCCCCTGAGTCAATGCC | 685 |
| 3. E815K | rs35845187 | Chr19:42475074 G/A | F: GCTGGACAGGCCGGGCGC  R: TCTGCTCCCCTGAGTCAATGCC | 928 |
|  | rs61355139 | Chr19:42474908 C/G | F: TGCCTGTAGTGCCAGCTACTCG  R: CGGCACTGGGTGGTAAGGAG | 634 |
|  | NA | Chr19:42475186 G/A | F: GGAGTCGAGGTTGCAGTGAGCT | 1044 |
|  |  |  | R: TCTGCTCCCCTGAGTCAATGCC |  |

**Table S4. Primers used for short tandem repeat (STR) analysis for paternity.**

| Marker | Primer (5’→3’) |
| --- | --- |
| D1S468 | F: AATTAACCGTTTTGGTCCT |
|  | R: GCGACACACACTTCCC |
| D3S1266 | F: AGATGAGGGGTAATGTTGGA |
|  | R: AAGCATCTTAATGGATGGAAA |
| D5S641 | F:AGTTGTGTATTGGAGAATGTTATCA |
|  | R: AGGGACAGTCCACTTCCAGT |
| D12S99 | F: GGCAGAAGTGCCTGGG |
|  | R: TCGAGGGTGCAGGTGG |
| D13S1265 | F: TACAGACCTCATAGGATTCTTT |
|  | R: TTGTTTTCTGCTAATGTGTG |
| D17S831 | F: CGCCTTTCCTCATACTCCAG |
|  | R: GCCAGACGGGACTTGAATTA |
| D22S1141 | F: GCTTCTCCACGAGCAAA |
|  | R: CTCCCCACAGCGTCAG |
| DXS 8075 | F: GGGCTACCAAAGGACTGT  R: CTGGGTTGTGACTGTTTCAT |
| DXS 6797 | F: TTCCCTCTCTCCCTCTGTCT  R: ACACACACCCAAAACCAGAT |
| DXS 6807 | F: GAGCAATGATCTCATTTGCA  R: AAGTAAACATGTATAGGAAAAAGCT |
| AR | F: TCCAGAATCTGTTCCAGAGCGTGC  R: GCTGTGAAGGTTGCTGTTCTCCAT |

| **Table S5. Primers used for PASM detection** | | | | |
| --- | --- | --- | --- | --- |
| Genomic position ^a^ | Variant^b^ | Forward | Reverse | Product size |
| Chr19:42479928 | NM_152296.4:c.2116G>A | TGACGATGGAGGCAAAGTTG | TGAGTCTGAGGGAGAAGGAG | 409 |
| Chr19:42474557 | NM_152296.4:c.2401G>A | GGCACTGGGTGGTAAGGAGA | CGCCTGATCTTCGACAACCT | 396 |
| Chr19:42474436 | NM_152296.4:c.2443G>A | GGCACTGGGTGGTAAGGAGA | CGCCTGATCTTCGACAACCT | 396 |
| Chr19:42473598 | NM_152296.4:c.2677G>A | ACAGACAGCAACTGGGCTTT | GGAATGATCCAGGCTCTCGG | 426 |
| Chr19:42471896 | NM_152296.4:c.2839G>C | GAGTTTCAGGGGACTGGAGG | GCAGGGTGGGTGCTCTC | 420 |
| Chr19:42471896 | NM_152296.4:c.2839G>A | GAGTTTCAGGGGACTGGAGG | GCAGGGTGGGTGCTCTC | 420 |
| Chr19:42486187_42486189 | NM_152296.4:c.1063_1065del | CTCACCTGACTGGTCCTCAG | TGTCTTTGGGTGTCTCTGCC | 396 |
| ^a^ Reference genome build: GRCh37.  ^b^ cDNA reference: NM_152296.4. | | | | |

| **Table S6. Summary for screening of variants in *ATP1A3*** | | | | | | | | | |
| --- | --- | --- | --- | --- | --- | --- | --- | --- | --- |
| Base substitution^a^ | Amino acid substitution^b^ | | Exon | Number of cases | Prediction by Polyphen2 | Prediction by iFish | | Publication | Hotspot |
| c.397A>C | p.(Thr133Pro) | | 5 | 1 | 1.0 | 0.9428 | | This study | No |
| c.410C>A | p.(Ser137Tyr) | | 5 | 1 | 1.0 | 0.8933 | | [[1](#_ENREF_1)] | No |
| c.829G>A | p.(Glu277Lys) | | 8 | 1 | 1.0 | 0.8868 | | [[2](#_ENREF_2)] | No |
| c.1013C>T | p.(Ala338Val) | | 9 | 1 | 1.0 | 0.8846 | | This study | No |
| c.1109C>A | p.(Thr370Asn) | | 9 | 1 | 1.0 | 0.9739 | | [[3](#_ENREF_3)] | No |
| c.1063_1065delGAA | p.(Glu355del) | | 9 | 1 |  | - | | This study | No |
| c.2116G>A | p.(Gly706Arg) | | 16 | 1 | 1.0 | 0.9431 | | [[4](#_ENREF_4)] | No |
| c.2263G>A | p.(Gly755Ser) | | 16 | 1 | 1.0 | 0.9254 | | [[5](#_ENREF_5)] | No |
| c.2263G>T | p.(Gly755Cys) | | 16 | 1 | 1.0 | 0.9493 | | [[6](#_ENREF_6)] | No |
| c.2312C>A | p.(Thr771Asn) | | 17 | 2 | 0.991 | 0.8462 | | [[5](#_ENREF_5)] | No |
| c.2312C>T | p.(Thr771Ile) | | 17 | 1 | 0.987 | 0.8016 | | [[5](#_ENREF_5)] | No |
| c.2316C>G | p.(Ser772Arg) | | 17 | 1 | 1.0 | 0.8238 | | [[5](#_ENREF_5)] | No |
| c.2318A>C | p.(Asn773Thr) | | 17 | 1 | 0.974 | 0.8399 | | [[5](#_ENREF_5)] | No |
| c.2401G>A | p.(Asp801Asn) | | 17 | 30 | 0.999 | 0.8945 | | [[7](#_ENREF_7)] | Yes |
| c.2403T>A | p.(Asp801Glu) | | 17 | 1 | 0.785 | 0.7854 | | [[8](#_ENREF_8)] | No |
| c.2405T>C | p.(Leu802Pro) | | 17 | 1 | 1.0 | 0.9634 | | [[5](#_ENREF_5)] | No |
| c.2413G>C | p.(Asp805His) | | 17 | 2 | 1.0 | 0.9383 | | [[5](#_ENREF_5)] | No |
| c.2417T>A | p.(Met806Lys) | | 17 | 1 | 0.837 | 0.7983 | | [[8](#_ENREF_8)] | No |
| c.2423C>T | p.(Pro808Leu) | | 18 | 1 | 1.0 | 0.9339 | | [[9](#_ENREF_9)] | No |
| c.2429T>A | p.(Ile810Asn) | | 18 | 1 | 1.0 | 0.9347 | | [[3](#_ENREF_3)] | No |
| c.2429T>C | p.(Ile810Thr) | | 18 | 1 | 1.0 | 0.9958 | | [[3](#_ENREF_3)] | No |
| c.2443G>A | p.(Glu815Lys) | | 18 | 18 | 1.0 | 0.9495 | | [[6](#_ENREF_6)] | Yes |
| c.2501T>C | p.(Leu834Ser) | | 18 | 2 | 1.0 | 0.9526 | | This study | No |
| c.2516T>C | p.(Leu839Pro) | | 18 | 2 | 1.0 | 0.9311 | | [[5](#_ENREF_5)] | No |
| c.2552A>C | p.(Gln851Pro) | | 18 | 1 | 0.995 | 0.9466 | | This study | No |
| c.2677G>A | p.(Gly893Arg) | | 19 | 2 | 1.0 | 0.8913 | | [[5](#_ENREF_5)] | No |
| c.2755_2757delGTC | p.(Val919del) | | 20 | 1 |  | - | | This study | No |
| c.2767G>A | p.(Asp923Asn) | | 20 | 3 | 0.998 | 0.7367 | | [[10](#_ENREF_10)] | No |
| c.2767G>T | p.(Asp923Tyr) | | 20 | 1 | 1.0 | 0.9497 | | [[3](#_ENREF_3)] | No |
| c.2781C>G | p.(Cys927Trp) | | 20 | 1 | 1.0 | 0.9154 | | [[3](#_ENREF_3)] | No |
| c.2839G>A | p.(Gly947Arg) | | 21 | 8 | 1.0 | 0.9241 | | [[5](#_ENREF_5)] | Yes |
| c.2839G>C | p.(Gly947Arg) | | 21 | 5 | 1.0 | 0.9434 | | [[5](#_ENREF_5)] | Yes |
| c.2974G>T | p.(Asp992Tyr) | | 21 | 2 | 1.0 | 0.9319 | | [[3](#_ENREF_3)] | No |
| Total |  | |  | 98 |  |  | |  |  |
| ^a^ cDNA reference: NM_152296.4.  ^b^Amino acid reference sequence: NP_689509.1. | | | | |  |  | |  |  |
| **Table S7. Parent-of-origin analysis by allele-specific PCR** | | | | | | | | | |
| Family | | Variation | | | | | Origin | | |
| A004 | | c.2429T>A | | | | | ND^a^ | | |
| A005 | | c.2443G>A | | | | | ND | | |
| A006 | | c.2312C>T | | | | | Maternal | | |
| A007 | | c.2443G>A | | | | | ND | | |
| A011 | | c.2401G>A | | | | | ND | | |
| A015 | | c.2443G>A | | | | | ND | | |
| A020 | | c.2401G>A | | | | | Paternal | | |
| A021 | | c.410C>A | | | | | Paternal | | |
| A022 | | c.2401G>A | | | | | Maternal | | |
| A023 | | c.2443G>A | | | | | ND | | |
| A024 | | c.2401G>A | | | | | ND | | |
| A025 | | c.2401G>A | | | | | ND | | |
| A026 | | c.2312C>A | | | | | Paternal | | |
| A028 | | c.2401G>A | | | | | Paternal | | |
| A031 | | c.2401G>A | | | | | Paternal | | |
| A033 | | c.2443G>A | | | | | Paternal | | |
| A035 | | c.2401G>A | | | | | Paternal | | |
| A036 | | c.2316C>G | | | | | Paternal | | |
| A037 | | c.2401G>A | | | | | ND | | |
| A039 | | c.2401G>A | | | | | Paternal | | |
| A040 | | c.2443G>A | | | | | Paternal | | |
| A042 | | c.2401G>A | | | | | ND | | |
| A046 | | c.2401G>A | | | | | Paternal | | |
| A048 | | c.2401G>A | | | | | ND | | |
| A052 | | c.2677G>A | | | | | ND | | |
| A054 | | c.2423C>T | | | | | Maternal | | |
| A056 | | c.2405T>C | | | | | Maternal | | |
| A059 | | c.2516T>C | | | | | Paternal | | |
| A060 | | c.2443G>A | | | | | Paternal | | |
| A061 | | c.2401G>A | | | | | Paternal | | |
| A062 | | c.2413G>C | | | | | Paternal | | |
| A063 | | c.2443G>A | | | | | Paternal | | |
| A064 | | c.2263G>T | | | | | Paternal | | |
| A065 | | c.2401G>A | | | | | Paternal | | |
| A074 | | c.2443G>A | | | | | ND | | |
| A076 | | c.2516T>C | | | | | Paternal | | |
| A077 | | c.2501T>C | | | | | Paternal | | |
| A079 | | c.2443G>A | | | | | Paternal | | |
| A080 | | c.2401G>A | | | | | Paternal | | |
| A086 | | c.2401G>A | | | | | Paternal | | |
| A087 | | c.2401G>A | | | | | ND | | |
| A089 | | c.2401G>A | | | | | Paternal | | |
| A091 | | c.2401G>A | | | | | Paternal | | |
| A092 | | c.397A>C | | | | | ND | | |
| A093 | | c.2401G>A | | | | | Paternal | | |
| A095 | | c.2401G>A | | | | | ND | | |
| A097 | | c.2401G>A | | | | | Paternal | | |
| A098 | | c.2413G>C | | | | | ND | | |
| A100 | | c.2401G>A | | | | | ND | | |
| A101 | | c.2443G>A | | | | | Paternal | | |
| A102 | | c.2443G>A | | | | | Paternal | | |
| A105 | | c.2443G>A | | | | | ND | | |
| A106 | | c.2443G>A | | | | | ND | | |
| A107 | | c.2403T>A | | | | | ND | | |
| A111 | | c.2401G>A | | | | | ND | | |
| ^a^ Unable to determine | | | | | | | | | |

**Table S8. Summary for patient variant information**

| Family | ID | Variation | Amino acid substitution | Publication | Paternal sperm collected and detected |
| --- | --- | --- | --- | --- | --- |
| A001 | A00103 | c.2839G>A | p.(Gly947Arg) | [[5](#_ENREF_5)] | no |
| A002 | A00203 | c.2839G>A | p.(Gly947Arg) | [[5](#_ENREF_5)] | no |
| A003 | A00303 | c.2417T>A | p.(Met806Lys) | [[5](#_ENREF_5)] | no |
| A004 | A00403 | c.2429T>A | p.(Ile810Asn) | [[5](#_ENREF_5)] | no |
| A005 | A00503 | c.2443G>A | p.(Glu815Lys) | [[5](#_ENREF_5)] | no |
| A006 | A00603 | c.2312C>T | p.(Thr771Ile) | [[5](#_ENREF_5)] | no |
| A007 | A00703 | c.2443G>A | p.(Glu815Lys) | [[5](#_ENREF_5)] | no |
| A011 | A01103 | c.2401G>A | p.(Asp801Asn) | [[5](#_ENREF_5)] | no |
| A012 | A01203 | c.2839G>C | p.(Gly947Arg) | [[5](#_ENREF_5)] | yes |
| A013 | A01303 | c.2443G>A | p.(Glu815Lys) | [[5](#_ENREF_5)] | no |
| A014 | A01403 | - | - | [[5](#_ENREF_5)] | no |
| A015 | A01503 | c.2443G>A | p.(Glu815Lys) | [[5](#_ENREF_5)] | yes |
| A016 | A01603 | c.2839G>A | p.(Gly947Arg) | [[5](#_ENREF_5)] | yes |
| A019 | A01903 | c.1109C>A | p.(Thr370Asn) | [[5](#_ENREF_5)] | no |
| A020 | A02003 | c.2401G>A | p.(Asp801Asn) | [[5](#_ENREF_5)] | yes |
| A021 | A02103 | c.410C>A | p.(Ser137Tyr) | [[5](#_ENREF_5)] | yes |
| A022 | A02203 | c.2401G>A | p.(Asp801Asn) | [[5](#_ENREF_5)] | no |
| A023 | A02303 | c.2443G>A | p.(Glu815Lys) | [[5](#_ENREF_5)] | yes |
| A024 | A02403 | c.2401G>A | p.(Asp801Asn) | [[5](#_ENREF_5)] | no |
| A025 | A02503 | c.2401G>A | p.(Asp801Asn) | [[5](#_ENREF_5)] | no |
| A026 | A02603 | c.2312C>A | p.(Thr771Asn) | [[5](#_ENREF_5)] | no |
| A027 | A02703 | c.2767G>T | p.(Asp923Tyr) | [[5](#_ENREF_5)] | no |
| A028 | A02803 | c.2401G>A | p.(Asp801Asn) | [[5](#_ENREF_5)] | no |
| A029 | A02903 | c.2839G>C | p.(Gly947Arg) | [[5](#_ENREF_5)] | no |
| A030 | A03003 | c.2839G>A | p.(Gly947Arg) | [[5](#_ENREF_5)] | yes |
| A031 | A03103 | c.2401G>A | p.(Asp801Asn) | [[5](#_ENREF_5)] | no |
| A032 | A03203 | c.2263G>A | p.(Gly755Ser) | [[5](#_ENREF_5)] | yes |
| A033 | A03303 | c.2443G>A | p.(Glu815Lys) | [[5](#_ENREF_5)] | yes |
| A035 | A03503 | c.2401G>A | p.(Asp801Asn) | [[5](#_ENREF_5)] | yes |
| A036 | A03603 | c.2316C>G | p.(Ser772Arg) | [[5](#_ENREF_5)] | yes |
| A037 | A03703 | c.2401G>A | p.(Asp801Asn) | [[5](#_ENREF_5)] | yes |
| A039 | A03903 | c.2401G>A | p.(Asp801Asn) | [[5](#_ENREF_5)] | no |
| A040 | A04003 | c.2443G>A | p.(Glu815Lys) | [[5](#_ENREF_5)] | yes |
| A041 | A04103 | c.2767G>A | p.(Asp923Asn) | [[5](#_ENREF_5)] | no |
| A042 | A04203 | c.2401G>A | p.(Asp801Asn) | [[5](#_ENREF_5)] | yes |
| A045 | A04503 | - | - | [[5](#_ENREF_5)] | no |
| A046 | A04603 | c.2401G>A | p.(Asp801Asn) | [[5](#_ENREF_5)] | yes |
| A048 | A04803 | c.2401G>A | p.(Asp801Asn) | [[5](#_ENREF_5)] | yes |
| A050 | A05003 | c.2116G>A | p.(Gly706Arg) | [[5](#_ENREF_5)] | yes |
| A052 | A05203 | c.2677G>A | p.(Gly893Arg) | [[5](#_ENREF_5)] | no |
| A053 | A05303 | c.2839G>A | p.(Gly947Arg) | [[5](#_ENREF_5)] | yes |
| A054 | A05403 | c.2423C>T | p.(Pro808Leu) | [[5](#_ENREF_5)] | no |
| A055 | A05503 | c.2839G>C | p.(Gly947Arg) | [[5](#_ENREF_5)] | yes |
| A056 | A05603 | c.2405T>C | p.(Leu802Pro) | [[5](#_ENREF_5)] | no |
| A058 | A05803 | - | - | [[5](#_ENREF_5)] | no |
| A059 | A05903 | c.2516T>C | p.(Leu839Pro) | [[5](#_ENREF_5)] | yes |
| A060 | A06003 | c.2443G>A | p.(Glu815Lys) | [[5](#_ENREF_5)] | yes |
| A061 | A06103 | c.2401G>A | p.(Asp801Asn) | [[5](#_ENREF_5)] | yes |
| A062 | A06203 | c.2413G>C | p.(Asp805His) | [[5](#_ENREF_5)] | no |
| A063 | A06303 | c.2443G>A | p.(Glu815Lys) | [[5](#_ENREF_5)] | yes |
| A064 | A06403 | c.2263G>T | p.(Gly755Cys) | [[5](#_ENREF_5)] | yes |
| A065 | A06503 | c.2401G>A | p.(Asp801Asn) | This study | yes |
| A066 | A06603 | c.2839G>C | p.(Gly947Arg) | This study | yes |
| A067 | A06703 | c.2839G>A | p.(Gly947Arg) | This study | yes |
| A068 | A06803 | - | - | This study | no |
| A069 | A06903 | c.2552A>C | p.(Gln851Pro) | This study | yes |
| A070 | A07003 | c.2501T>C | p.(Leu834Ser) | This study | no |
| A071 | A07103 | c.2677G>A | p.(Gly893Arg) | This study | yes |
| A072 | A07203 | c.2974G>T | p.(Asp992Tyr) | This study | yes |
| A073 | A07303 | c.2443G>A | p.(Glu815Lys) | This study | no |
| A074 | A07403 | c.2443G>A | p.(Glu815Lys) | This study | yes |
| A075 | A07503 | c.2767G>A | p.(Asp923Asn) | This study | no |
| A076 | A07603 | c.2516T>C | p.(Leu839Pro) | This study | yes |
| A077 | A07703 | c.2501T>C | p.(Leu834Ser) | This study | yes |
| A078 | A07803 | - | - | This study | no |
| A079 | A07903 | c.2443G>A | p.(Glu815Lys) | This study | yes |
| A080 | A08003 | c.2401G>A | p.(Asp801Asn) | This study | yes |
| A081 | A08103 | c.829G>A | p.(Glu277Lys) | This study | yes |
| A082 | A08203 | c.2839G>C | p.(Gly947Arg) | This study | yes |
| A083 | A08303 | - | - | This study | no |
| A084 | A08403 | - | - | This study | no |
| A085 | A08503 | c.2839G>A | p.(Gly947Arg) | This study | no |
| A086 | A08603 | c.2401G>A | p.(Asp801Asn) | This study | yes |
| A087 | A08703 | c.2401G>A | p.(Asp801Asn) | This study | yes |
| A088 | A08803 | c.2781C>G | p.(Cys927Trp) | This study | no |
| A089 | A08903 | c.2401G>A | p.(Asp801Asn) | This study | no |
| A090 | A09003 | c.2318A>C | p.(Asn773Thr) | This study | no |
| A091 | A09103 | c.2401G>A | p.(Asp801Asn) | This study | no |
| A092 | A09203 | c.397A>C | p.(Thr133Pro) | This study | yes |
| A093 | A09303 | c.2401G>A | p.(Asp801Asn) | This study | yes |
| A094 | A09403 | c.2443G>A | p.(Glu815Lys) | This study | no |
| A095 | A09503 | c.2401G>A | p.(Asp801Asn) | This study | yes |
| A096 | A09603 | c.2839G>A | p.(Gly947Arg) | This study | yes |
| A097 | A09703 | c.2401G>A | p.(Asp801Asn) | This study | yes |
| A098 | A09803 | c.2413G>C | p.(Asp805His) | This study | no |
| A099 | A09903 | c.1013C>T | p.(Ala338Val) | This study | no |
| A100 | A10003 | c.2401G>A | p.(Asp801Asn) | This study | no |
| A101 | A10103 | c.2443G>A | p.(Glu815Lys) | This study | yes |
| A102 | A10203 | c.2443G>A | p.(Glu815Lys) | This study | yes |
| A103 | A10303 | c.2974G>T | p.(Asp992Tyr) | This study | no |
| A104 | A10403 | c.2443G>A | p.(Glu815Lys) | This study | no |
| A105 | A10503 | c.2443G>A | p.(Glu815Lys) | This study | yes |
| A106 | A10603 | c.2443G>A | p.(Glu815Lys) | This study | no |
| A107 | A10703 | c.2403T>A | p.(Asp801Glu) | This study | no |
| A108 | A10803 | c.2312C>A | p.(Thr771Asn) | This study | yes |
| A111 | A11103 | c.2401G>A | p.(Asp801Asn) | This study | yes |
| A112 | A11203 | c.1063_1065del | p.(Glu355del) | This study | yes |
| A113 | A11303 | c.2755_2757del | p.(Val919del) | This study | no |
| A115 | A11503 | c.2401G>A | p.(Asp801Asn) | This study | no |
| A116 | A11603 | c.2429T>C | p.(Ile810Thr) | This study | no |
| A117 | A11703 | c.2767G>A | p.(Asp923Asn) | This study | no |
| A118 | A11803 | c.2401G>A | p.(Asp801Asn) | This study | no |
| A119 | A11903 | c.2401G>A | p.(Asp801Asn) | This study | no |
| A120 | A12003 | c.2401G>A | p.(Asp801Asn) | This study | no |
| A123 | A12303 | c.2401G>A | p.(Asp801Asn) | This study | no |

**References:**

1. Novy J, McWilliams E, Sisodiya SM. Asystole in alternating hemiplegia with de novo ATP1A3 mutation. *Eur J Med Genet* 2014;57:37-39.

2. Boelman C, Lagman-Bartolome AM, MacGregor DL, et al. Identical ATP1A3 mutation causes alternating hemiplegia of childhood and rapid-onset dystonia parkinsonism phenotypes. *Pediatr Neurol* 2014;51:850-853.

3. Brashear A, Sweadner K, Cook J, et al. ATP1A3-Related Neurologic Disorders GeneReview Table 2. 2014.

4. Hully M, Ropars J, Hubert L, et al. Mosaicism in ATP1A3-related disorders: not just a theoretical risk. *Neurogenetics* 2016;18:23-28.

5. Yang X, Gao H, Zhang J, et al. ATP1A3 mutations and genotype-phenotype correlation of alternating hemiplegia of childhood in Chinese patients. *PLoS One* 2014;9:e97274.

6. Ishii A, Saito Y, Mitsui J, et al. Identification of ATP1A3 mutations by exome sequencing as the cause of alternating hemiplegia of childhood in Japanese patients. *PLoS One* 2013;8:e56120.

7. Brashear A, Dobyns WB, de Carvalho Aguiar P, et al. The phenotypic spectrum of rapid-onset dystonia-parkinsonism (RDP) and mutations in the ATP1A3 gene. *Brain* 2007;130:828-835.

8. Heinzen EL, Arzimanoglou A, Brashear A, et al. Distinct neurological disorders with ATP1A3 mutations. *Lancet Neurol* 2014;13:503-514.

9. Sasaki M, Ishii A, Saito Y, et al. Progressive Brain Atrophy in Alternating Hemiplegia of Childhood. *Movement Disorders Clinical Practice* 2017;4:406-411.

10. Anselm IA, Sweadner KJ, Gollamudi S, et al. Rapid-onset dystonia-parkinsonism in a child with a novel atp1a3 gene mutation. *Neurology* 2009;73:400-401.
